# Electroacupuncture treatment can improve cognitive impairment in spontaneously hypertensive rats: a preliminary DTI study

**DOI:** 10.3389/fnins.2025.1637037

**Published:** 2025-08-28

**Authors:** Ji-peng Liu, Bing-xuan Han, Yu Liu, Bin-bin Nie, Tao Bian, Chuan Liu, Tian-qi Xia, Yu Gong, Long-teng Tu, Jing Zhang, Bing-hui Wang, Yi Yang, Song-Li Li, Lin-ding He, Qing-guo Liu, Meng Xu

**Affiliations:** 1 School of Acupuncture-Moxibustion and Tuina, Beijing University of Chinese Medicine, Beijing, China; 2 College of Special Education, Beijing Union University, Beijing, China; 3 Institute of High Energy Physics, Chinese Academy of Sciences, Beijing, China; 4 Rulin Community Health Center, Beijing, China; 5 Wangjing Community Health Center, Beijing, China; 6 Department of Tuina, Beijing University of Chinese Medicine Third Affiliated Hospital, Beijing, China

**Keywords:** hypertension, impairment of cognitive function, electroacupuncture, diffusion tensor imaging (DTI), white matter structure, SHRs

## Abstract

**Background:**

Hypertension is a significant risk factor for cognitive impairment. Our group’s previous rs-fMRI study has found that acupuncture can enhance the functional connectivity of brain regions related to cognitive function, thereby protecting the cognitive function of spontaneously hypertensive rats (SHRs).

**Methods:**

This study aimed to reveal the effects of electroacupuncture on the white matter structure in the brain regions of SHRs using the diffusion tensor imaging (DTI) technique. 20 SHRs were divided into the electroacupuncture group (EA) and the model group (SHR), and 10 Wistar-Kyoto rats were established as the normal control group (WKY). Electroacupuncture was applied to Taichong (LR3) and Zusanli (ST36) acupoints for 12 weeks, with treatment every other day. Blood pressure was measured once every 2 weeks, with DTI scans and the Morris water maze (MWM) tests performed at the end of the 12-week electroacupuncture intervention.

**Results:**

The results showed that electroacupuncture significantly decreased systolic and diastolic blood pressure and enhanced spatial learning and memory in SHRs. DTI analysis revealed that hypertension led to increased axial diffusivity (AD), mean diffusivity (MD), and radial diffusivity (RD) values in brain regions such as the hippocampus, prefrontal cortex, striatum, amygdaloid body, posterior lobe of cerebellum, olfactory bulb, and piriform cortex, indicating white matter microstructural damage. Electroacupuncture improved these injuries, especially significantly improving the integrity of the hippocampal white matter structure. Correlation analysis showed that hippocampal white matter structure parameters were significantly correlated with behavioral manifestations of MWM.

**Conclusion:**

Therefore, we speculate that electroacupuncture can alleviate white matter damage in the hippocampus, prefrontal cortex, striatum, and other brain regions, thereby preventing cognitive impairment in SHRs, which may be one of the reasons for the efficacy of electroacupuncture.

## 1 Introduction

Hypertension is one of the most common chronic diseases, and it is also a significant risk factor for target organ damage, including the brain (Zhou et al., 2021). Long-term hypertension impairs brain structure and function (Pires et al., 2013; Rizzoni et al., 2009; Santisteban et al., 2023), which in turn affects cognitive functions such as executive function, motor neuron activity, and attention in patients (Theofilis et al., 2024). These cognitive impairments significantly increase the incidence of vascular dementia and Alzheimer’s disease (Cheon, 2022). Currently, more than 9.5 million vascular dementia cases worldwide are attributed to uncontrolled hypertension (Hughes et al., 2020), and the prevalence of mild cognitive impairment (MCI) is higher in hypertensive patients than in non-hypertensive patients (Qin et al., 2021). Several studies have also shown that the correlation between hypertension and cognition is age-dependent, with midlife hypertension associated with an increased risk of vascular dementia and Alzheimer’s disease in later life (Freitag et al., 2006; Hughes et al., 2020; Wei et al., 2018). Therefore, early blood pressure management is significant for reducing cognitive impairments. Major US blood pressure guidelines suggest that lowering blood pressure is a strategy reasonable for preventing cognitive decline (Heidenreich et al., 2022). Studies have reported modest improvements in cognitive function in hypertensive patients treated with commonly used antihypertensive medications (Hajjar et al., 2020; Hanon et al., 2008; Igase et al., 2012; Tzourio et al., 1999). However, these medications have many contraindications, poor treatment compliance, and side effects (Dzau and Hodgkinson, 2024; Mahmood et al., 2021; Vrijens et al., 2008). Acupuncture therapy, as a representative intervention of traditional medicine, is highly popular due to its unique advantages of fewer side effects and a high safety profile (Wang et al., 2023; Yu et al., 2021). Several randomized controlled trials have shown that acupuncture not only lowers blood pressure and reduces blood pressure fluctuations (Fan et al., 2023; Lin et al., 2023; Zhang et al., 2021; Zhou et al., 2024) but also exerts protective effects against hypertension-induced brain injury (Sun et al., 2014; Cui et al., 2024). The group’s previous studies in several animal experiments confirmed that acupuncture can achieve blood pressure regulation in spontaneously hypertensive rats (SHRs) through multi-system, multi-target, and multi-level mechanisms of action (Li et al., 2023; Qiulei et al., 2018; Xie et al., 2015; Yang et al., 2022) while also alleviating brain injury and protecting cognitive function (Liu et al., 2023). However, the mechanisms underlying acupuncture’s neuroprotective effects and cognitive improvement remain poorly understood, highlighting the need for further research.

Diffusion tensor imaging (DTI), was developed based on diffusion-weighted imaging (DWI) and is used as a noninvasive neuroimaging technique mainly for the observation and tracking of cerebral white matter conduction tracts, with the advantages of stereoscopy, directionality, and high accuracy (Kantarci et al., 2017; Nanda et al., 2022). The core principle is to reconstruct the direction and integrity of nerve fibers through diffusion differences of water molecules in anisotropic tissues such as white matter fibers (Latini et al., 2022; Min et al., 2020). DTI characterizes white matter damage through several parameters, including mean diffusivity (MD), which indicates the overall degree of water molecule dispersion, with an increase suggesting disruption of tissue integrity (Song et al., 2002). Axial diffusivity (AD) reflects the ability to diffuse along the direction of the axon, which is highly correlated with axonal damage (Budde et al., 2009). Radial diffusivity (RD) is perpendicular to the direction of fiber travel, and its increase tends to suggest myelin loss (Wheeler-Kingshott and Cercignani, 2009). DTI is uniquely suited for detecting hypertension-related cerebral small vessel disease and cognitive impairment, allowing for the early detection of microstructural damage that conventional imaging cannot detect (de Laat et al., 2011; Mendez Colmenares et al., 2024). Studies have shown that MD is highly sensitive to microstructure (Haddad et al., 2022) and can detect early signs of structural changes in brain white matter (Dykan et al., 2020; Wartolowska & Webb, 2021). Elevated MD values are associated with decreased executive function and information processing speed (de Groot et al., 2013), AD values are commonly increased in older hypertensive patients with subjective memory impairment; and elevated RD values are suggestive of myelin damage, which is strongly associated with attentional and working memory deficits (Fletcher et al., 2013; Sala et al., 2016). In addition, white matter structural aberrations have been demonstrated in the cingulate, hippocampus, uncinate fasciculus, and corpus callosum in brain regions of cognitively impaired patients (Gold et al., 2012; Nowrangi et al., 2013; Teipel et al., 2010), and the degree of cognitive impairment has been shown to have a statistically significant correlation with an increase in the MD value (Clerx et al., 2012; Finkelstein et al., 2021; Mayo et al., 2019).

DTI technology has also been gradually applied to explore the mechanism of acupuncture in recent years, enabling a more intuitive examination of changes in brain white matter during acupuncture from the perspective of brain structure. Through DTI testing, it was found that acupuncture can enhance cognitive impairment by remodeling the microstructure of patients’ memory-related brain regions. The study found that acupuncture can protect the cognitive function of rats with vascular dementia by decreasing the RD and AD values (Ma et al., 2020). Another study (Wang et al., 2017) found the change of parameter values before and after treatment of patients with cognitive impairment was measured by using DTI technology, and found that the apparent diffusion coefficient (ADC) values of the frontal lobe, knee and pressure of the corpus callosum on both sides of the patient decreased, indicating that the white matter structure had changed in the early stage of the disease, and acupuncture can promote the repair of white matter function, thereby improving cognitive function.

To the best of our knowledge, few studies have been reported to explore the protective effects of electricity on cognitive function in hypertension using the DTI technique. Therefore, SHRs were chosen as the experimental model in this study, and we evaluated the efficacy of electroacupuncture in lowering blood pressure and the effectiveness of cognitive function protection in SHRs by blood pressure measurement and the Morris water maze (MWM) test data analysis. We also used the MD, AD, and RD parameters of DTI to observe the differences in the white matter structure of brain regions in SHRs, to explore the relationship between the different brain regions and cognitive function, to find the target brain regions activated or protected by electroacupuncture, and reveal the neuroimaging mechanism of acupuncture for lowering blood pressure and protecting cognitive function in SHRs, which will provide a reference for the selection of the target brain regions in the future study.

## 2 Materials and methods

### 2.1 Animals

20 male SHRs (14 weeks old, 280 g ± 20 g) and 10 male Wistar-Kyoto rats (WKYs, 14 weeks old, 280 g ± 20 g) were supplied by Beijing Vital River Laboratory Animal Technology Co., Ltd., (SCXK [Beijing] 2016–0006). All rats were required to adapt to the feeding environment 1 week before the experiment. They were housed in an SPF-grade laboratory at Beijing University of Traditional Chinese Medicine, where the animals lived in an environment with a temperature of 24 ± 1°C, a humidity of 55 ± 5°C, a light–dark cycle of 12 h, lights turned on at 8:00 am every day, and feed and water supplied for 24 h. The rats were kept in the Beijing University of Traditional Chinese Medicine laboratory. All animal experiments were approved by the Animal Care and Use Committee of the Beijing University of Chinese Medicine (BUCM-4-2021041001-2093).

### 2.2 Experimental procedures

**Figure 1:**
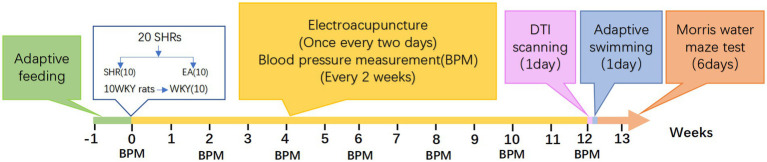
Experimental process.

The experimental design is depicted in Figure 1. After all rats were acclimatized and fed for 1 week, 20 SHRs were randomly divided into two groups according to the random number table method: the model group (SHR, *n* = 10), the electroacupuncture group (EA, *n* = 10), and 10 WKYs were set up as a normal control group (WKY, *n* = 10). All rats received intervention once every other day for 12 consecutive weeks, and blood pressure was measured every 2 weeks. At the end of the 12 weeks, eight rats in each group were randomly selected to undergo DTI scans, and the MWM test was performed after the scan. Then, all rats were anesthetized and executed.

### 2.3 Blood pressure measurement

Two experienced technicians measured tail artery blood pressure in rats at the room temperature of 23 ± 2°C at fixed times from 8:00 am to 11:00 am on the 0th, 14th, 28th, 42nd, 56th, 70th, and 84th days of the experimental cycle. Each unanesthetized rat was placed in a blood pressure measuring mouse set (with a thermostat to keep the temperature constant at 36°C) and warmed up for 10 min. Then, blood pressure was measured in the caudal artery of the rats using a small animal blood pressure measuring system (BP-2010E, Softron Biotechnology, Beijing, China). Blood pressure was measured three times in each rat, and the average of the three blood pressures was taken as the measurement result. To ensure the accuracy and reproducibility of the measurements, the rats were trained once a day for 1 week before the experiment to acclimatize to both the rat tail immobilization facility and the measurement environment.

### 2.4 Interventional methods

**Figure 2:**
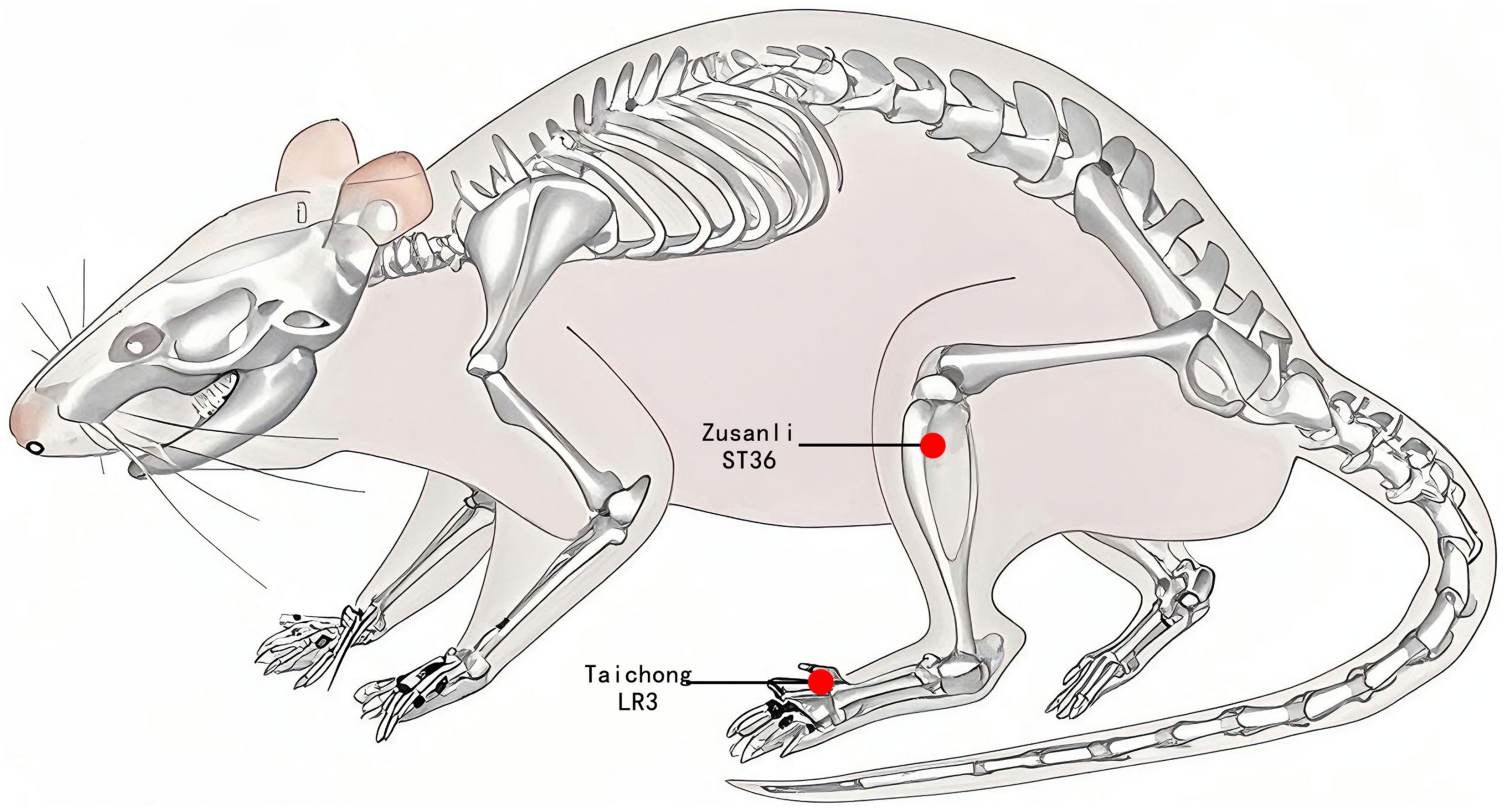
Acupuncture point location. The red dot indicates the location of Zusanli acupoint (ST36) and Taichong acupoint (LR3).

In the EA group, disposable sterile stainless-steel needles (0.18 × 13 mm, ZHONGYANTAIHE, Beijing, China) were used to puncture the rats bilaterally at the bilateral Taichong acupoints (LR3) at an inclined angle of 30 degrees to a depth of 2 mm, as well as at the bilateral Zusanli acupoints (ST36) at a vertical angle to a depth of 5 mm, as illustrated in Figure 2. Two electrodes were connected to LR3 and ST36 on the same side. Both sides of the acupoints were simultaneously connected to an acupoint nerve stimulator (Gensun Medical Technology, Jiangsu, China) for current stimulation at a strength of 1 mA/2 Hz, with each treatment lasting 20 min. The SHR and WKY groups did not receive treatment, and the same grasping and immobilizing stimulation was applied for 20 min each time. Interventions were performed every other day from 1:00 to 4:00 pm, with a total intervention cycle of 12 weeks.

### 2.5 The MWM test

The MWM test was divided into two parts: the localization navigation experiment and the spatial exploration experiment, with the experiments conducted daily from 8:00 am to 12:00 pm for a total of 6 days. One day before the formal experiment, the rats were placed in a pool (without a platform) for adaptive swimming (60 s) to familiarize themselves with the environment. We divided the cylindrical pool (160 cm in diameter and 50 cm in depth) into four quadrants and positioned the circular platform (10 cm in diameter) about 1 to 2 cm below the surface of the target quadrant. In the localization and navigation experiment, the time it took the rats to find the platform (evasion latency) from entering the water in different quadrants was recorded, with a time limit of 60 s. If the time limit was exceeded, the time was recorded as 60 s. The rats were placed on the platform for 10 s to help them acclimate to the surrounding environment. Each rat underwent training four times a day, with a 30-min interval between each session, for five consecutive days, and the average escape latency for each rat was calculated for each day. Spatial exploration experiments were performed on day six, with one test per rat. After removing the platform, we observed and recorded the time spent by the rat in the initial target quadrant within 60 s, the number of times it crossed the platform area, and its exploration trajectory.

### 2.6 DTI

#### 2.6.1 Anesthesia and fixation of laboratory animals

Eight rats from each group were randomly selected for DTI scanning. Before the scanning, the rats were anesthetized with a mixture of 5% isoflurane and 95% oxygen. Additionally, the rats were injected dexmedetomidine hydrochloride (100 μg/mL) into the lateral muscle of the hind limb of rats, with a dosage of 0.02 mL per 100 g of body weight. The rats were immobilized on a special scanning bed in the prone position during the scan. They wore masks to inhale a mixture of 2% isoflurane and 98% oxygen to maintain anesthesia. A physiological monitor continuously tracked the rats’ body temperature, respiratory rate, and heart rate in real-time (Model 1,025, Small Animal Instruments Inc., Stony Brook, NY, United States).

#### 2.6.2 Scanning sequence and parameter settings

All subject rats were scanned using the 7.0 T Bruker animal *in vivo* MRI scanner (PharmaScan 70/16 US, Bruker, Germany) with a small animal-specific head surface coil. First, the T2_TurboRARE sequence was used to acquire T2-weighted images with parameters set as follows: repetition time (TR) = 5,500 ms, echo time (TE) = 33 ms, percent phase field view = 100, slice thickness = 0.5 mm, acquisition matrix = 256 × 256, and flip angle = 90°. Next, the rat brain’s DTI was performed using a single-shot echo planar imaging (EPI) sequence. The parameters were: TE = 34 ms, TR = 3,280 ms, flip angle = 90°, slice thickness = 0.3 mm, number of averages = 1, percent phase field view = 100, and acquisition matrix = 128 × 80.

#### 2.6.3 Image processing

The DTI image analysis process involved data preprocessing, quantitative calculation, and statistical analysis. First, the original DICOM data were converted to NIFTI format and quality screened to ensure data integrity and the absence of significant artifacts. Subsequently, the origin of each rat’s brain image was unified to the intracranial center through image origin correction to maintain spatial consistency. In the preprocessing stage, the FSL tool was utilized to perform head motion correction, eddy current correction, and gradient correction, eliminating image distortion caused by motion artifacts and magnetic field inhomogeneity. Diffusion parameters, including AD, MD, and RD, were calculated using DTI_TOOLKIT to quantify the microstructural features of brain white matter. For cross-subject comparison, the rat brain probability map (Liang et al., 2017) developed by the research group was used as the spatial standard from the spmratIHEP toolkit, and the quantitative indicators were spatially normalized using the DARTEL algorithm to ensure alignment of the data in a unified coordinate system. Gaussian smoothing of the spatially normalized data was performed using a half-height width of [4 4 4] (standard after expanding the voxel size) to remove noise even further and to make the statistically analyzed data conform to a normal distribution. Based on the rat Paxinos brain atlas, the AD, MD, and RD values of each brain region were extracted from the normalized images, enlarged 1,000 times, and saved to enhance the accuracy of data presentation. Finally, the entire brain was statistically analyzed pixel by pixel through a generalized linear model combined with a two-sample t-test. The significance threshold (*p* < 0.001) and cluster threshold were set at > 20 voxels to generate a heatmap of intergroup differences, where warm colors indicate regions of parameter increase and cool colors indicate regions of parameter decrease, visually presenting brain region-specific changes.

### 2.7 Statistical analysis

The experimental data collected during the experiments were analyzed using SPSS 20.0 software (IBM, Armonk, NY, United States). This software was first used to test for normality and then for homogeneity of variance. Two-way repeated measurement analysis of variance was performed on the experimental data regarding rat systolic and diastolic blood pressure, as well as escape latency in the WMW test. A one-way analysis of variance was conducted on the time spent in the target quadrant, the number of crossings over the escape platform, and the AD, MD, and RD values of the hippocampus, using Fisher’s least significant difference (LSD) test for pairwise comparisons between groups. A significance level of *p* < 0.05 was considered statistically significant. The DTI data were modeled using a general linear model. Data were analyzed using one-way ANOVA, and a *post hoc* two-sample t-test was performed, in which AD, MD, and RD values were considered statistically significant at *p* < 0.001 (uncorrected) and clusters > 20 voxels. Pearson (for data conforming to normal distribution) or Spearman (for data not conforming to normal distribution) methods were used to analyze correlations between the AD, MD, and RD values of brain regions in the WKY, SHR, and EA groups and behavioral indices, including the number of crossing the platform and the duration of time spent in the target quadrant. A significance level of *p* < 0.05 was considered statistically significant. The line and bar charts in the experiment were plotted by Graphpad Prism 8 (Graphpad Software, San Diego, CA, United States).

## 3 Results

### 3.1 Electroacupuncture significantly lowered blood pressure in SHRs

**Figure 3:**
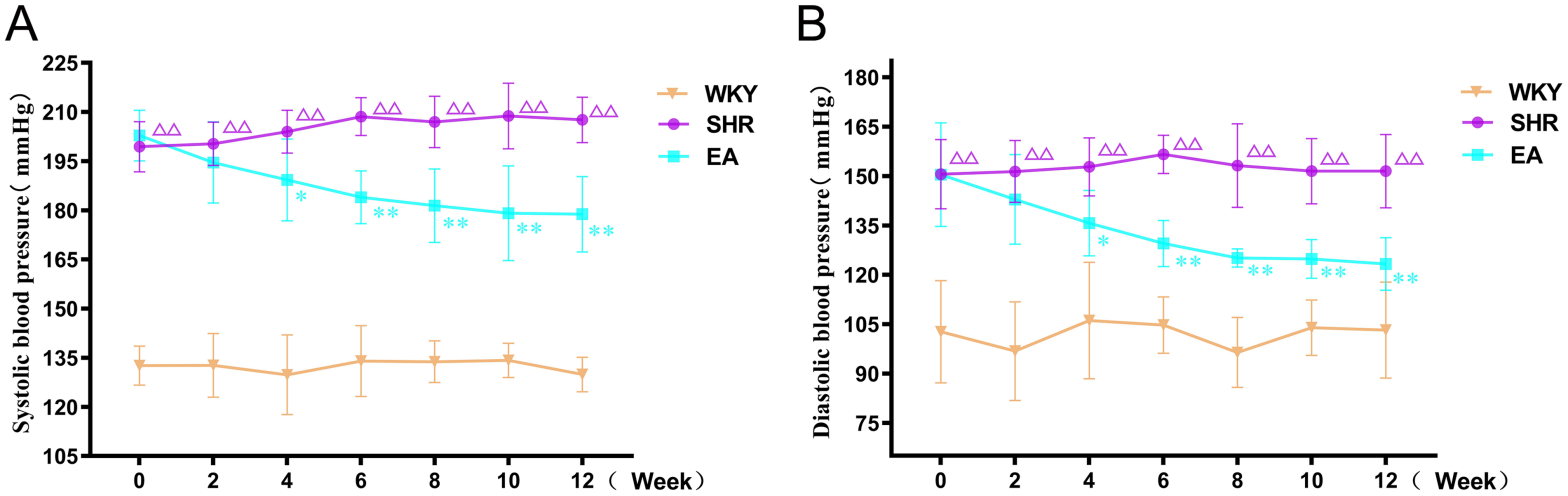
Systolic and diastolic blood pressure measurements in each group. **(A)** Systolic blood pressure measurement. **(B)** Diastolic blood pressure measurements. Values are expressed as mean ± SD (*n* = 10 for each group). ^ΔΔ^*p* < 0.01 vs. the WKY group; *p < 0.05 and **p < 0.01 vs. the SHR group. WKY, the normal control group; SHR, the model group; EA, the electroacupuncture group.

As shown in Figure 3, there was a significant treatment effect (*F* = 796.045, *p* < 0.01), a time effect (*F* = 1.425, *p* < 0.01), and an interaction between treatment and time (*F* = 5.656, *p* < 0.01) in the analysis of systolic blood pressure data in rats. Similarly, there was a significant therapeutic effect (*F* = 179.712, *p* < 0.01) and time effect (*F* = 3.414, *p* < 0.01) in the diastolic blood pressure data of rats, and there was an interaction between the treatment effect and the time effect (*F* = 4.364, *p* < 0.01). This suggested that each group’s systolic blood pressure and diastolic blood pressure showed significant differences at different periods respectively, indicating that the blood pressures of the SHRs changed continuously with acupuncture treatment. Compared to the WKY group, systolic and diastolic blood pressures in the SHR group gradually increased and tended to stabilize at higher levels during weeks 0–12 of the intervention (*p* < 0.01). Compared to the SHR group, systolic blood pressure in the EA group began to decrease at week 4 of measurement (*p* < 0.05), and continued to decrease until the 12th week (both *p* < 0.01). Similarly, diastolic blood pressure in the EA group began to decrease at week 4 of measurement compared with the SHR group (*p* < 0.05), and continued to decrease until the 12th week (both *p* < 0.01). The findings demonstrated that electroacupuncture significantly reduced systolic and diastolic blood pressure in spontaneously hypertensive rats (SHRs), exhibiting a time-cumulative effect on blood pressure regulation.

### 3.2 Electroacupuncture can protect the cognitive function of SHRs

**Figure 4:**
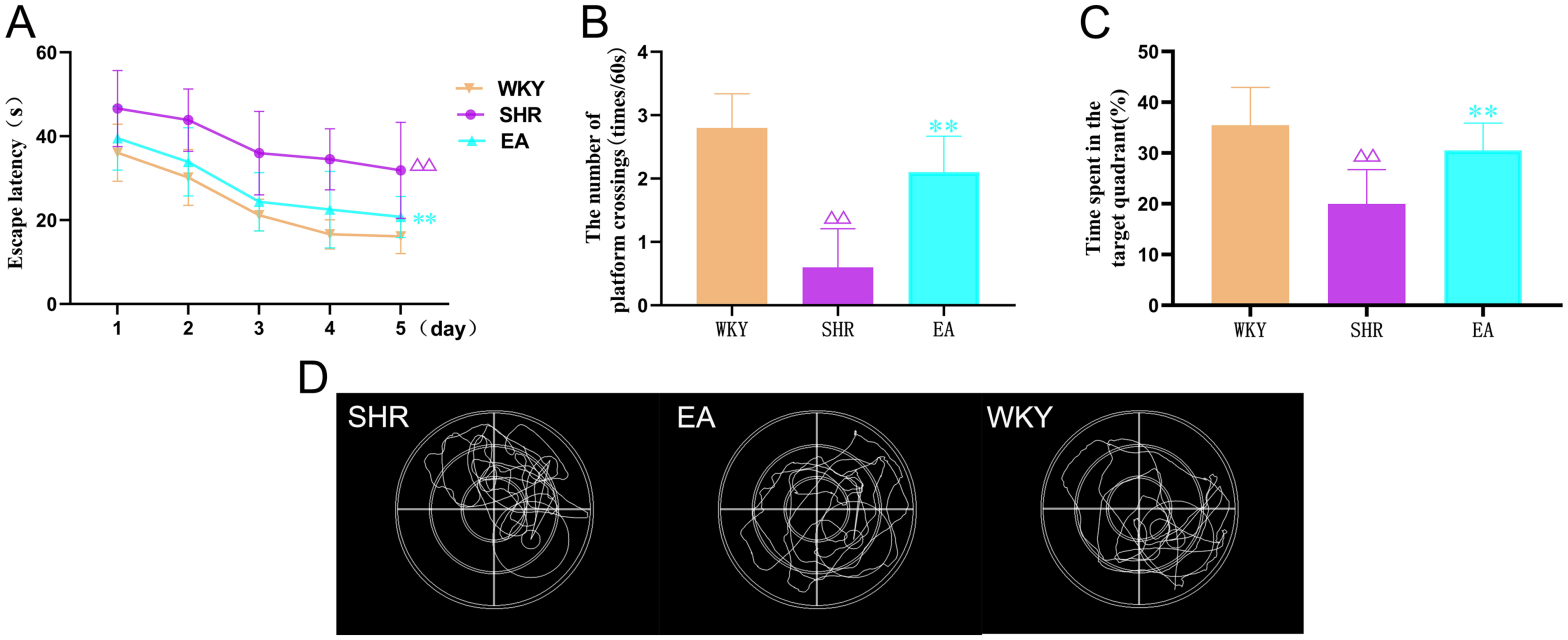
**(A)** The results of the escape latency for rats in each group. **(B)** The results show the number of times each group crossed the escape platform. **(C)** Time spent results in target quadrants for each group. **(D)** Typical swimming trajectories of rats in the detection test. Values are expressed as mean ± SD (*n* = 10 for each group). ^ΔΔ^*p* < 0.01 vs. the WKY group; **P < 0.01 vs. the SHR group. WKY, the normal control group; SHR, the model group; EA, the electroacupuncture group.

MWM can reflect the spatial learning memory function in rats. The analysis revealed a significant treatment effect (*F* = 43.096, *p* < 0.001) and time effect (*F* = 33.479, *p* < 0.001) for escape latency in the acquired training experiment (Figure 4A). The SHR group required more time to find hidden escape platforms than the WKY group (*p* < 0.01). In contrast, the EA group required significantly less time than the SHR group (*p* < 0.01). In the exploration experiment (Figures 4B–D), the time spent in the target quadrant and the number of times crossing the escape platform were significantly lower in the SHR group compared to the WKY group (both *p* < 0.01). The time spent on the target quadrant and the number of crossings of the escape platform were significantly higher in the EA group compared with the SHR group (both *p* < 0.01). The results suggested that long-term hypertension caused impairment of learning and memory function, and electroacupuncture had a protective effect on cerebral cognitive impairment in hypertension.

### 3.3 Electroacupuncture can modulate the white matter structure of SHRs

**Figure 5:**
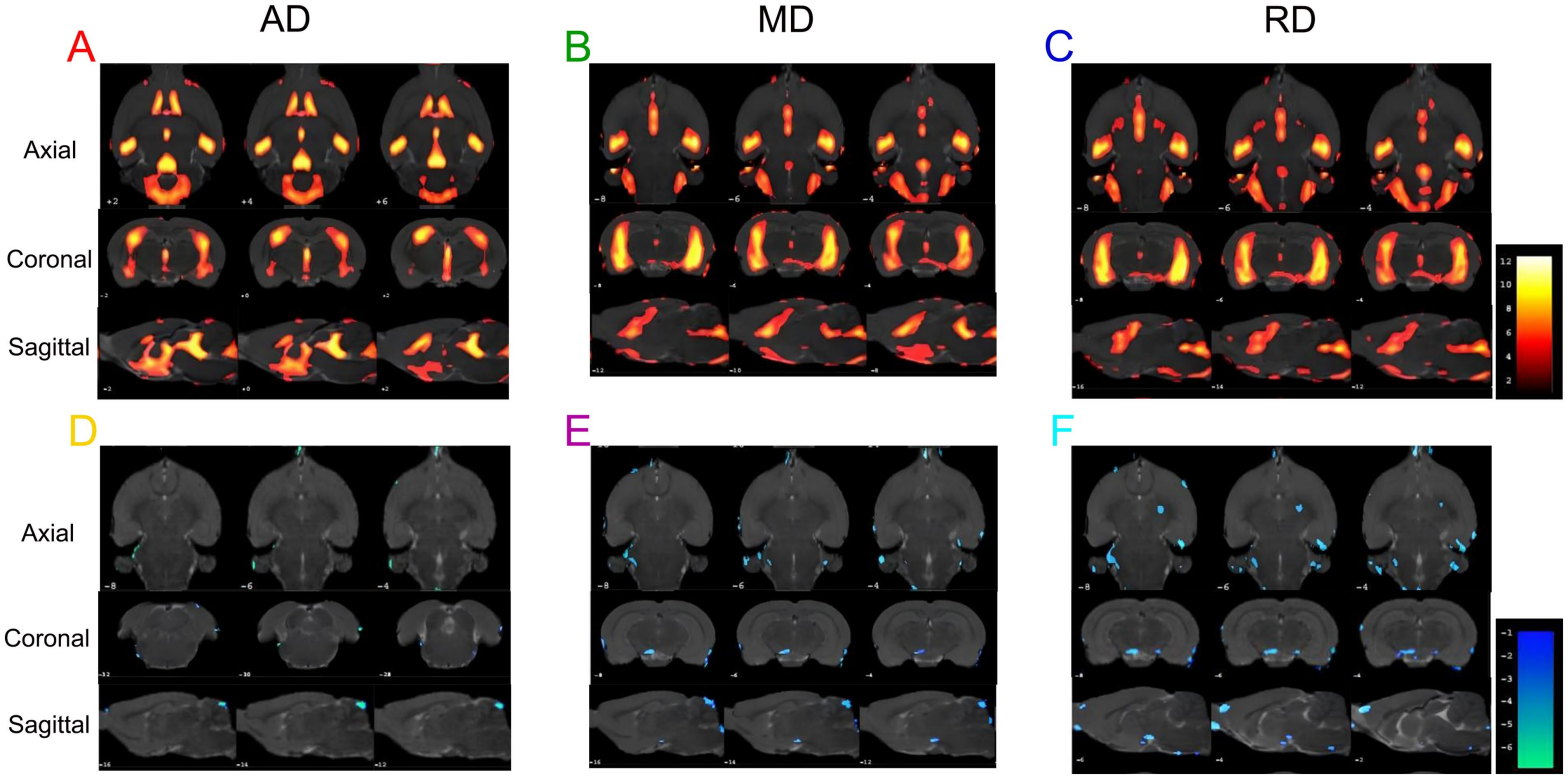
The results of the AD, MD, and RD values are different in each group. **(A)** Brain regions with increased AD values in the SHR group compared to the WKY group. **(B)** Brain regions with increased MD values in the SHR group compared to the WKY group. **(C)** Brain regions with increased RD values in SHR compared to the WKY group. **(D)** Brain regions with decreased AD values in the EA group compared to the SHR group. **(E)** Brain regions with decreased MD values in the EA group compared to the SHR group. **(F)** Brain regions with decreased RD values in the EA group compared to the SHR group. Differential brain regions are displayed sequentially on the axial, coronal, and sagittal planes (*p* < 0.001, uncorrected, clusters > 20 voxels, *n* = 8 for each group). The color bars are used to signify the t-value of the group analysis; the color is brighter, indicating a higher t-value.

We analyzed the AD, MD, and RD for DTI scanning. Compared to the WKY group, the SHR group showed significantly increased AD, MD, and RD values in some brain regions (Figures 5A–C; Supplementary Tables 1–3), indicating that long-term hypertension damaged the white matter structure in some brain regions. Consistently, compared with the SHR group, AD, MD, and RD values were significantly decreased in some brain regions of the EA group (Figures 5D-F; Supplementary Tables 4–6), indicating that electroacupuncture regulated the white matter structure in these brain regions.

### 3.4 Seek out the focus of electroacupuncture interventions in the brain region

**Figure 6:**
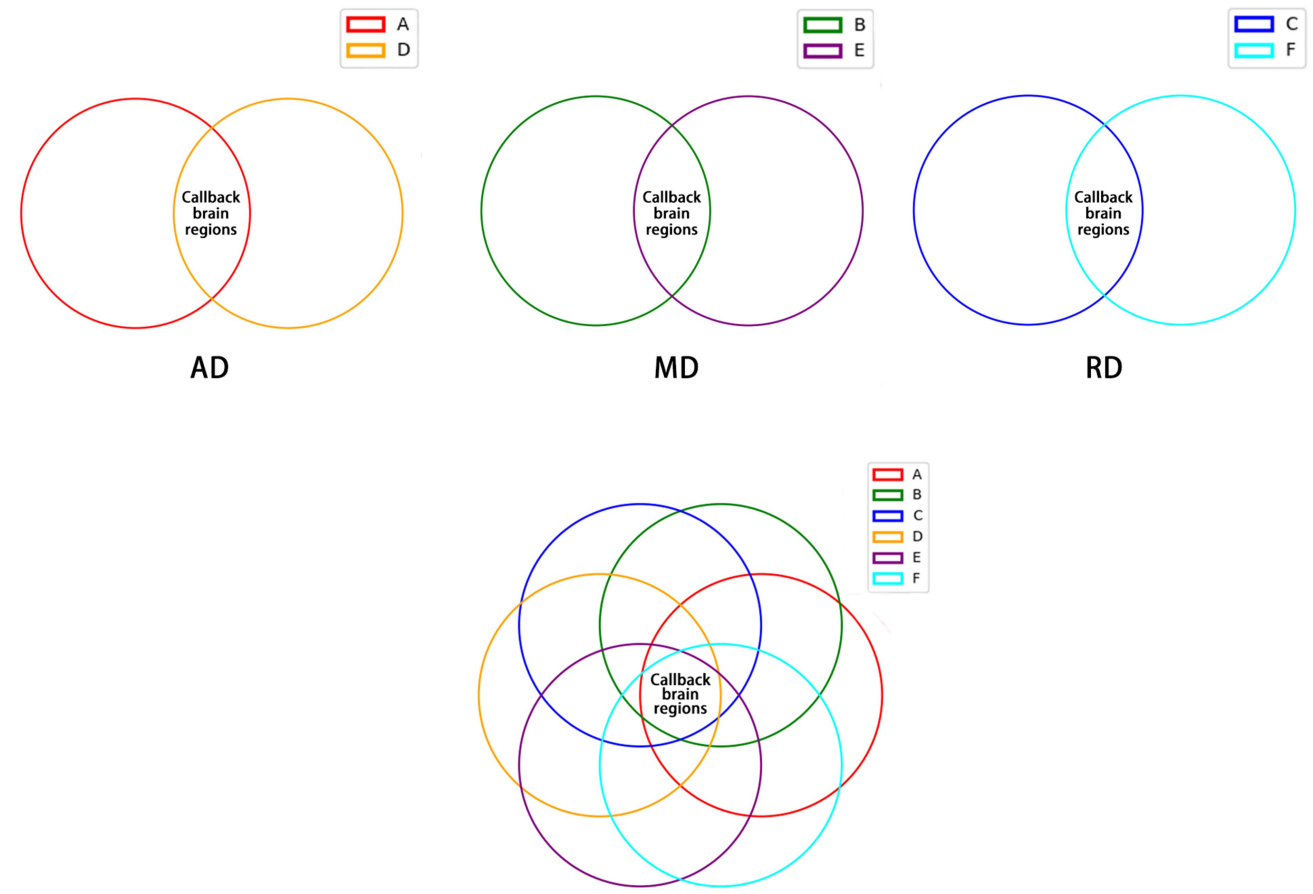
The callback brain regions common to AD, MD, and RD values. **(A)** Brain regions with increased AD values in the SHR group compared to the WKY group. **(B)** Brain regions with increased MD values in the SHR group compared to the WKY group. **(C)** Brain regions with increased RD values in SHR compared to the WKY group. **(D)** Brain regions with decreased AD values in the EA group compared to the SHR group. **(E)** Brain regions with decreased MD values in the EA group compared to the SHR group. **(F)** Brain regions with decreased RD values in the EA group compared to the SHR group.

As shown in Figure 6, 32 callback brain regions of electroacupuncture were obtained by intersecting the brain regions with increased AD values in the SHR group compared to the WKY group, and the brain regions with decreased AD values in the EA group compared to the SHR group (Supplementary Table 7). In the same method, 64 callback brain regions of electroacupuncture were obtained by MD value (Supplementary Table 8), and 80 callback brain regions of electroacupuncture were obtained by RD value (Supplementary Table 9). After that, we again intersected the above-mentioned callback brain regions that were valued by AD, MD, and RD and obtained a total of 28 focused callback brain regions in which electroacupuncture affected AD, MD, and RD values, including hippocampus (left and right), striatum (left and right), prefrontal cortex (right), posterior lobe of cerebellum (left), olfactory bulb (left), and piriform cortex (right), amygdaloid body (right; Supplementary Table 10). Most of these callback brain regions were related to regulating blood pressure and cognitive function. It is also a key target brain region for electroacupuncture intervention, and electroacupuncture can effectively improve white matter damage in these brain regions.

### 3.5 Electroacupuncture can improve hippocampal white matter structure damage in SHRs

**Figure 7:**
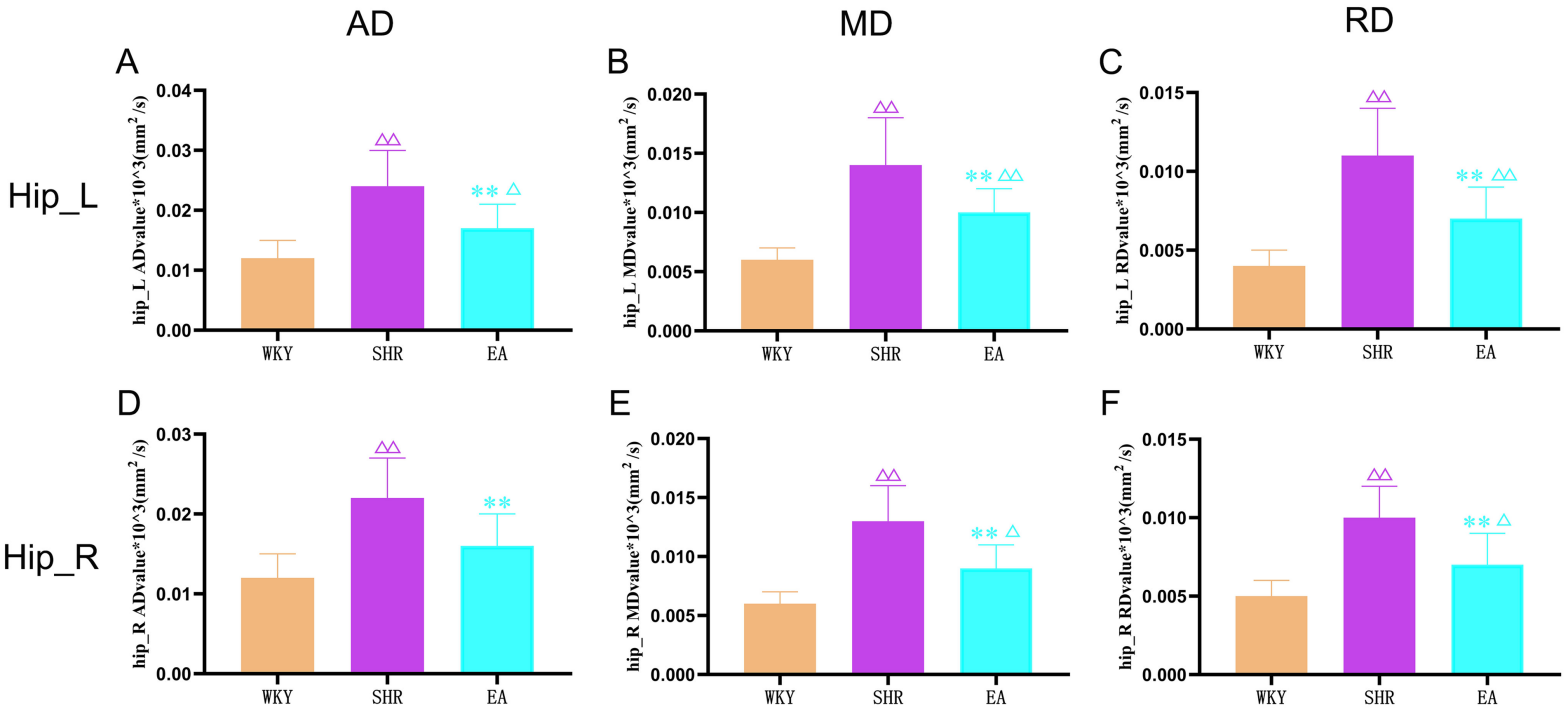
The results of the left and right hippocampal white matter indicators for each group. **(A)** Analysis results of AD values in the left hippocampus of each group. **(B)** Analysis results of MD values in the left hippocampus of each group. **(C)** Analysis results of RD values in the left hippocampus of each group. **(D)** Analysis results of AD values in the right hippocampus of each group. **(E)** Analysis results of MD values in the right hippocampus of each group. **(F)** Analysis results of RD values in the right hippocampus of each group. Values are expressed as mean ± SD (*n* = 8 for each group). ^Δ^*p* < 0.05 and ^ΔΔ^*P* < 0.01 vs. the WKY group; **p < 0.01 vs. the SHR group. Hip_L, the left hippocampus; Hip_R, the right hippocampus.; WKY, the normal control group; SHR, the model group; EA, the electroacupuncture group.

As shown in Figure 7, compared to the WKY group, the AD, MD, and RD values of the left and right hippocampal white matter were increased significantly in the SHR group (both *p* < 0.01). The AD, MD, and RD values of the left and right hippocampal white matter were decreased significantly in the EA group rats compared to the SHR group (both *p* < 0.01). Moreover, compared with the WKY group, the AD values of the right hippocampus in the EA group were not statistically significant (*p* > 0.05), indicating that electroacupuncture effectively improved the structural damage to the hippocampal white matter.

### 3.6 Correlation analysis of white matter indicators and WMW indicators in the hippocampus

**Figure 8:**
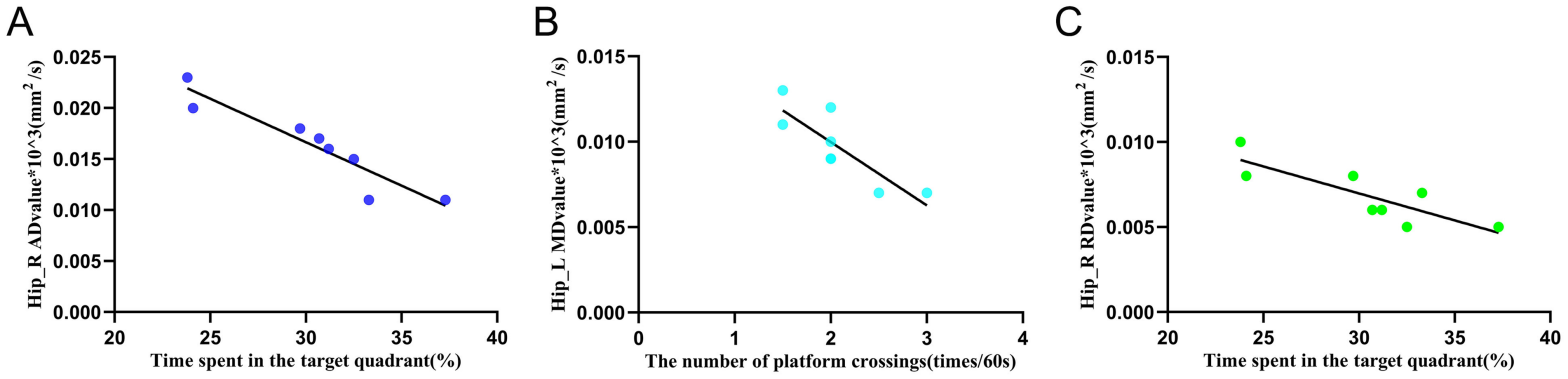
Relationship between white matter indicators and behavioral manifestations of the water maze in the left and right hippocampus. **(A)** Correlation analysis between the AD value of the right hippocampus and the spent time in the target quadrant in the EA group. **(B)** Correlation analysis between the MD value of the left hippocampus and the number of platform crossings in the EA group. **(C)** Correlation analysis between the RD value of the right hippocampus and the time spent in the target quadrant in the EA group. Pearson correlation analysis expresses the mean ± SD (*n* = 8 for each group), *p* < 0.05 is statistically significant, and *r* is the correlation coefficient. Hip_L, the left hippocampus; Hip_R, the right hippocampus.

The results of the Pearson correlation analysis (Figures 8A–C) showed that there was a significant correlation between the AD value of the right hippocampus in the EA group and the residence time in the target quadrant in the WMW test (*r* = −0.936, *p* = 0.001). There was a significant correlation between the MD value of the left hippocampus in the EA group and the number of crossings of the escape platform in the WMW test (*r* = −0.84, *p* = 0.009). There was a significant correlation between the RD value of the right hippocampus in the EA group and the spent time in the target quadrant (*r* = −0.836, *p* = 0.01). Additionally, they all showed a negative correlation, suggesting that the more intact the hippocampal white matter structure was under the electroacupuncture intervention, the better the rats performed in the WMW test.

## 4 Discussion

In clinical and experimental studies, LR3 and ST36 are commonly used acupuncture points for reducing blood pressure. Previous research has demonstrated that acupuncture at the LR3 and ST36 has a positive effect on both the immediate and long-term management of blood pressure (Yang et al., 2022; Zhang et al., 2021). The results of this study confirmed that the electroacupuncture intervention significantly lowered both systolic and diastolic blood pressure in SHRs, with the antihypertensive effect showing a time-cumulative effect. It was essential to note that the antihypertensive effect of electroacupuncture began at the fourth week and continued to decrease thereafter, which is consistent with findings reported in previous studies of electroacupuncture that modulate the hypothalamic-autonomic nervous system to achieve sustained hypotensive reduction (Zhang et al., 2022; Zhou et al., 2024). Furthermore, this study found that the decrease in both systolic and diastolic blood pressure in the EA group occurred simultaneously, which may be related to the fact that electroacupuncture can alleviate hypertension by improving white matter perfusion and fiber integrity (Ma et al., 2020).

SHRs are affected by long-term hypertension, leading to a gradual decline in cognitive performance (Gannon et al., 2023; Tayebati et al., 2012). It has been shown that SHRs develop stable hypertension by 12 weeks of age and cognitive impairment by 26–28 weeks of age (Johnson et al., 2020). We aimed to explore the preventive and protective effects of electroacupuncture on cognitive impairment related to hypertension, so 14-week-old SHRs were chosen as the test subjects. Their blood pressure was stable, and they were in the early stages of cognitive impairment. Previous studies from our group have demonstrated that electroacupuncture not only regulates blood pressure in SHRs but also enhances cognitive function by improving functional activity and connectivity in brain regions (Liu et al., 2023). The MWM test in this study further confirmed that the escape latency of SHRs in the EA group was decreased significantly compared to the SHR group, while their time spent in the target quadrant and the number of crossings increased, indicating that electroacupuncture effectively improves spatial learning and memory impairment. This finding aligns with the protective effect of blood pressure control on cognitive function suggested by clinical studies (Hajjar et al., 2020; Lennon et al., 2023).

It is well known that DTI is currently the only noninvasive imaging method that can assess the structural integrity of white matter *in vivo* (Im et al., 2018). When a cranial brain lesion occurs, the microstructural integrity of the brain tissue is disrupted, and the diffusion anisotropy of water molecules in the tissue is altered, resulting in a change in the values of DTI parameters (Min et al., 2020). In this study, we found that compared with the rats in the WKY group, the AD, RD, and MD values of several brain regions in SHRs were differentially increased. This result showed that long-term hypertension can lead to damage to cerebral white matter structures, which may be due to vascular endothelial dysfunction, disruption of the blood–brain barrier, or neuroinflammatory correlations induced by long-term hypertension (Pires et al., 2013; Sala et al., 2016; Wang et al., 2019). AD values typically indicate axonal integrity, and increased levels may be linked to hypertension-related vascular endothelial dysfunction or axonal swelling (Maier-Hein et al., 2017; Zhang et al., 2019); an increase in RD values is suggestive of structural damage to the myelin sheaths, which may originate from hypertension-induced cerebral vasculopathy and ischemic injury (Pantoni, 2010; Song et al., 2002); and an overall increase in MD values further supports extensive damage to brain tissue microstructure (Basser et al., 1994). These changes may precede lesions visible on conventional imaging (e.g., white matter hyperintensities or lacunar infarcts), suggesting that DTI parameters (AD, RD, and MD) may serve as sensitive biomarkers of early brain damage in hypertension (Joutel et al., 2010; Promjunyakul et al., 2018).

Several DTI experimental studies have shown that objective changes in brain white matter structure after acupuncture intervention can intuitively reveal the mechanism of action of acupuncture efficacy in the pathological state of the disease (Liu et al., 2024; Zhao et al., 2018; Han et al., 2024; Gao, 2020), but there are few studies on the mechanism of acupuncture intervention in hypertensive brain injury reported. In this study, we found that electroacupuncture affected AD, MD, and RD values in some brain regions of SHRs. After analyzing intergroup metrics, we found that electroacupuncture can protect against damage to the white matter structure in brain regions related to blood pressure and cognition. The brain regions involved were the hippocampus, prefrontal cortex, striatum, amygdaloid body, and posterior lobe of cerebellum, etc. The hippocampus plays a crucial role in learning and memory (Lisman et al., 2017), and its reduced functional connectivity and associated memory impairment have been linked to hypertension (Feng et al., 2020). The hippocampus belongs to the limbic system along with the amygdaloid body and striatum, which is involved in the regulation of human emotions, behavior, memory, olfaction, and the biological clock, with the striatum having an essential role in decision-making, reward processing, inhibitory control, and task switching (Haber, 2016), and its iron deposition partially mediating the relationship between hypertension and cognitive function (Gao et al., 2025); while the amygdaloid body can eliminate fear memories with the intervention of antihypertensive drugs (Rahnemayan et al., 2025); the prefrontal cortex is responsible for higher-level cognitive functions (e.g., working memory, social behavior), and contains fibrous structures such as the corpus callosum, superior longitudinal bundle, and cingulate bundle to connect with other brain regions to ensure that prefrontal cortex can integrate sensory, memory, emotion, and other information to make higher-level decisions (Coley et al., 2021; Yamasaki et al., 2002). Clinical studies have demonstrated that activation of the prefrontal cortex positively impacts executive abilities in hypertensive patients (Bishnoi et al., 2021). In addition, a growing number of studies have found that atrophy of the posterior lobe of cerebellum is associated with the progression of AD disease (Arleo et al., 2024), and in particular, with the increased vascular risk of hypertension, the posterior lobe of cerebellum involved in cognition may be activated to compensate for cortical deficits (Bai et al., 2011). Therefore, in the present study, we speculate that electroacupuncture protects the white matter structure in the hippocampus, prefrontal cortex, striatum, posterior lobe of cerebellum, and other brain regions, which in turn play an essential role in blood pressure lowering and protection of cognitive function. Interestingly, this study also found increased AD, MD, and RD values in the olfactory bulb and piriform cortex in the SHR group compared to the WKY group and decreased AD, MD, and RD values in the olfactory bulb and piriform cortex in the EA group compared to the SHR group. Previous studies have shown that the olfactory bulb and the piriform cortex are two of the most dominant brain regions in the olfactory system (Linster and Cleland, 2016), suggesting that hypertension causes damage to brain regions related to olfaction, and that electroacupuncture also has a protective effect against damage to their white matter structure. This result is consistent with our group’s previous research finding that acupuncture can activate the functional activity of brain regions related to olfactory function (Liu et al., 2023), which suggests that we should also pay attention to olfactory changes in hypertensive patients in the context of hypertensive cognitive impairment.

The hippocampus has long been at the center of research on memory and cognition. Studies have shown that it is primarily responsible for encoding, consolidating, and retrieving situational memories (Eichenbaum, 2017) and storing long-term memories by interacting with cortical networks. In addition, the hippocampus is involved in spatial navigation (O’Keefe and Dostrovsky, 1971) and the simulation of future scenarios (Schacter et al., 2008). The structural integrity of its white matter plays a vital role in performing executive and memory functions (Lacalle-Aurioles and Iturria-Medina, 2023). Therefore, we analyzed the hippocampal region’s AD, MD, and RD values in the WKY, SHR, and EA groups. The study showed that hypertension increased the AD, MD, and RD values in the hippocampus of SHRs, suggesting damage to the white matter structure. In contrast, the AD, MD, and RD values of the EA group decreased in the hippocampus of SHRs, thereby improving the structural integrity of the white matter in this region. In addition, we found that the parameter values of the hippocampal region in the EA group were significantly correlated with the indicators of the MWM test, suggesting that the structural integrity of the hippocampal white matter can influence the performance of SHRs in the WMW test and further confirming that the performance of SHRs in cognitive function tests is related to the hippocampus. Our group’s previous study found impaired hippocampal functional activity in SHRs by functional magnetic resonance imaging. Still, electroacupuncture did not ameliorate the impaired hippocampal functional activity (Liu et al., 2023), suggesting that our electroacupuncture’s effect on the structure of the hippocampal white matter may precede its impact on the functional activity of the hippocampus. Therefore, detecting the structural integrity of hippocampal white matter has a positive impact on the early diagnosis of cognitive impairment and provides a reference for the imaging diagnosis of mental impairment caused by hypertension.

There are several limitations of this study. As a neuroimaging study, this experiment mainly focuses on the macroscopic changes in the brain. It explores the effects of electroacupuncture on the white matter of brain regions. In future studies, we will explore the molecular mechanisms of the structural effects of acupuncture on white matter in combination with proteomics and genomics. Secondly, SHRs were chosen as a model of cognitive impairment in this experiment, and we could not guarantee that each SHR showed cognitive impairment. Therefore, we focused on the protective effect of acupuncture on the cognitive function of SHRs by comparing the EA group and the SHR group at the end of the intervention. Finally, the clinical relevance of the experimental model in this study was slightly lower than that of hypertensive patients. However, it should be noted that other underlying conditions, such as hyperglycemia, often accompany hypertensive patients presenting with cognitive impairment. SHRs suffer from a single disease, and the mechanism study is clearer; we will further investigate the mechanism of action of acupuncture to improve cognitive function in patients with hypertension.

## 5 Conclusion

In summary, electroacupuncture effectively reduces systolic and diastolic blood pressure in SHRs and has a protective effect on cognitive function in SHRs. The results of DTI analysis showed that electroacupuncture reduced AD, RD, and MD values of white matter in the hippocampus, prefrontal cortex, striatum, amygdaloid body, posterior lobe of cerebellum, olfactory bulb, and piriform cortex, and other brain regions related to the modulation of blood pressure and cognitive function, and thus improve the white matter damage in the related brain regions. It may be one of the reasons why electroacupuncture prevents cognitive impairment caused by hypertension.

## Data Availability

The datasets presented in this study can be found in online repositories. The names of the repository/repositories and accession number(s) can be found in the article/Supplementary material.
