# Correction: Electroacupuncture treatment can improve cognitive impairment in spontaneously hypertensive rats: a preliminary DTI study

**DOI:** 10.3389/fnins.2026.1821556

**Published:** 2026-03-13

**Authors:** Ji-peng Liu, Bing-xuan Han, Yu Liu, Bin-bin Nie, Tao Bian, Chuan Liu, Tian-qi Xia, Yu Gong, Long-teng Tu, Jing Zhang, Bing-hui Wang, Yi Yang, Song-Li Li, Lin-ding He, Qing-guo Liu, Meng Xu

**Affiliations:** 1 School of Acupuncture-Moxibustion and Tuina, Beijing University of Chinese Medicine, Beijing, China; 2 College of Special Education, Beijing Union University, Beijing, China; 3 Institute of High Energy Physics, Chinese Academy of Sciences, Beijing, China; 4 Rulin Community Health Center, Beijing, China; 5 Wangjing Community Health Center, Beijing, China; 6 Department of Tuina, Beijing University of Chinese Medicine Third Affiliated Hospital, Beijing, China

**Keywords:** hypertension, impairment of cognitive function, electroacupuncture, diffusion tensor imaging (DTI), white matter structure, SHRs

There was a mistake in Figure 1 as published. The two instances of the number 12 in the image should be corrected to the number 10. The corrected Figure 1 appears below.

**Figure 1:**
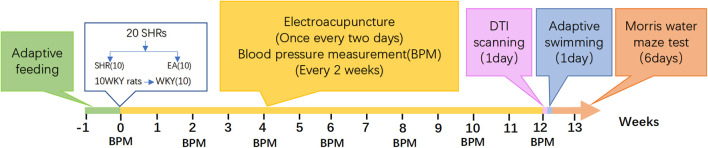
Experimental process.

The original version of this article has been updated.

