# Supplementary material for: Electroacupuncture treatment can improve cognitive impairment in spontaneously hypertensive rats: a preliminary DTI study
Source: Front Neurosci. 2025 Aug 28;19:1637037. doi: 10.3389/fnins.2025.1637037 (PMC12423048; doi:10.3389/fnins.2025.1637037)
Supplement: Supplementary file 1 [file Data_Sheet_1.docx]

Supplementary Material

Electroacupuncture Treatment can Improve Cognitive Impairment in Spontaneously Hypertensive Rats: A preliminary DTI Study

Ji-peng Liu^1✝^, Bing-xuan Han^1✝^, Yu Liu^2^, Bin-bin Nie^3^, Tao Bian^4^, Chuan Liu^5^, Tian-qi Xia^5^, Yu Gong^1^, Long-teng Tu^1^, Jing Zhang^1^, Bing-hui Wang^1^, Yi Yang^1^, Song-Li Li^1^, Lin-ding He^1^, Qing-guo Liu^1*^, Meng Xu^6*^

* Correspondence:

Qing-guo Liu: liuqingguo888@vip.sina.com

Meng Xu: chilli.xu@163.com

**Table S1. Brain regions with increased AD values in the SHR group compared to those in the WKY group.**

| Brain regions | Ke | *t*-value | Peak MNI coordinate (mm) | | |
| --- | --- | --- | --- | --- | --- |
|  |  |  | X | Y | Z |
| Thalamus_lateral nucleus group_left | 359 | 6.1413 | -4.3433 | 6.5489 | -5.1579 |
| Thalamus_lateral nucleus group_right | 453 | 7.8954 | 4.2029 | 7.3438 | -5.1579 |
| Thalamus_medial nucleus group_left | 335 | 8.4561 | -0.0042 | 5.7705 | -9.9579 |
| Thalamus_medial nucleus group_right | 117 | 6.7422 | -0.0108 | 6.312 | -9.4779 |
| Thalamus_midline nucleus group_left | 222 | 6.2684 | -0.3998 | 6.74 | -1.3179 |
| Thalamus_midline nucleus group_right | 128 | 6.5489 | 0.0047 | 6.8056 | -1.7979 |
| accumbens nucleus_left | 80 | 5.338 | -1.4815 | 7.3759 | 0.6021 |
| accumbens nucleus_right | 229 | 5.3715 | 0.6332 | 7.4137 | 1.5621 |
| amygdaloid body_left | 47 | 7.2986 | -0.0108 | 5.1438 | -9.4779 |
| amygdaloid body_right | 27 | 8.5941 | -0.0108 | 6.0111 | -9.4779 |
| anterior commissure_left | 26 | 4.6908 | -1.4749 | 7.7194 | 0.1221 |
| anterior commissure_right | 50 | 5.0329 | 0.5212 | 7.877 | -0.3579 |
| anterior nucleus group _left | 49 | 6.1788 | -0.2548 | 6.2065 | -1.7979 |
| anterior nucleus group _right | 16 | 5.4932 | 0.0047 | 7.3897 | -1.7979 |
| auditory cortex_left | 71 | 7.7197 | -6.0708 | 5.4075 | -6.5979 |
| auditory cortex_right | 102 | 7.7266 | 5.5548 | 6.3804 | -6.5979 |
| bed nucleus of stria terminalis _left | 62 | 5.4545 | -1.6133 | 7.4105 | 0.1221 |
| bed nucleus of stria terminalis _right | 84 | 5.2589 | 0.5212 | 8.1602 | -0.3579 |
| capsule_left | 111 | 5.0157 | -3.2969 | 7.1855 | -13.3179 |
| capsule_right | 90 | 4.516 | 3.2425 | 7.8467 | -13.3179 |
| cerebellum _anterior lobe of cerebellum _left | 1298 | 9.07 | -0.4153 | 5.0782 | -8.9979 |
| cerebellum _anterior lobe of cerebellum _right | 1375 | 9.0796 | 0.5189 | 5.1322 | -8.9979 |
| cerebellum _cerebellar nucleus_left | 263 | 6.9245 | -1.4219 | 5.7254 | -13.7979 |
| cerebellum _cerebellar nucleus_right | 233 | 6.2049 | 1.5191 | 5.8954 | -13.7979 |
| cerebellum _posterior lobe of cerebellum_left | 2117 | 7.7605 | -1.0902 | 3.7101 | 1.0821 |
| cerebellum _posterior lobe of cerebellum_right | 1631 | 7.8861 | 0.5146 | 3.5864 | 0.1221 |
| cerebral peduncle _left | 1 | 3.9044 | -3.952 | 7.6975 | -4.6779 |
| cerebral peduncle _right | 6 | 5.2552 | 3.7984 | 8.1455 | -4.6779 |
| cingulate gyrus _left | 701 | 9.18 | -0.4087 | 5.8465 | -9.4779 |
| cingulate gyrus _right | 906 | 9.1162 | 0.3739 | 4.9223 | -8.5179 |
| claustral layer _left | 9 | 4.2943 | -1.7913 | 6.8637 | 3.0021 |
| corpus callosum _left | 662 | 9.2315 | -5.1498 | 5.0931 | -5.6379 |
| corpus callosum _right | 639 | 9.9689 | 5.5416 | 5.9943 | -5.6379 |
| dentate gyrus_left | 373 | 8.4312 | -5.2882 | 6.5365 | -5.6379 |
| dentate gyrus_right | 364 | 9.8742 | 5.1437 | 7.1395 | -5.6379 |
| dorsal peduncular cortex_right | 5 | 4.5018 | 0.62 | 7.0453 | 2.5221 |
| fourth ventricle _left | 52 | 5.0554 | -1.0836 | 6.9564 | 0.6021 |
| fourth ventricle _right | 51 | 5.2355 | -0.0217 | 7.3609 | 0.1221 |
| hippocampus_left | 1314 | 9.1255 | -5.1498 | 5.3763 | -5.6379 |
| hippocampus_right | 1190 | 10.6967 | 5.4032 | 7.1545 | -5.6379 |
| hypothalamus_preoptic region_left | 107 | 5.4836 | -0.0966 | 8.0946 | -3.2379 |
| hypothalamus_preoptic region_right | 141 | 5.0753 | 0.7914 | 7.8504 | 0.1221 |
| hypothalamus_tuberal region _left | 301 | 5.3844 | -1.5537 | 9.7071 | -14.2779 |
| hypothalamus_tuberal region _right | 227 | 4.9981 | -0.0834 | 8.1798 | -4.1979 |
| infralimbic cortex_left | 6 | 3.9013 | -0.8505 | 6.3762 | 2.5221 |
| infralimbic cortex_right | 1 | 3.8065 | -0.1758 | 6.4152 | 2.5221 |
| insular cortex_left | 12 | 4.2929 | -3.4881 | 8.2881 | -9.4779 |
| interstitial nucleus _left | 7 | 4.0277 | -2.268 | 9.4125 | -11.3979 |
| interstitial nucleus _right | 7 | 4.4825 | 2.1435 | 9.6675 | -11.3979 |
| mammillary region_left | 43 | 5.6073 | -0.09 | 8.1372 | -3.7179 |
| mammillary region_right | 43 | 5.3473 | 0.0311 | 8.1442 | -3.7179 |
| medulla oblongata_left | 669 | 5.8281 | -0.6395 | 7.5785 | -2.7579 |
| medulla oblongata_right | 473 | 5.4458 | -0.1032 | 7.8927 | -2.7579 |
| midbrain _ inferior colliculus _left | 147 | 8.8209 | -0.4153 | 5.6623 | -8.9979 |
| midbrain _ inferior colliculus _right | 131 | 8.608 | 0.5189 | 5.4154 | -8.9979 |
| midbrain _ periaqueductal gray matter_left | 13 | 6.9735 | -0.1624 | 5.9179 | -8.5179 |
| midbrain _ periaqueductal gray matter_right | 4 | 6.4715 | 0.0971 | 5.9329 | -8.5179 |
| midbrain_tegmentum of midbrain_left | 137 | 6.1255 | 1.6336 | 5.5766 | -13.3179 |
| midbrain_tegmentum of midbrain_right | 263 | 6.1375 | -0.0108 | 4.8429 | -9.4779 |
| motor cortex _left | 1013 | 9.0065 | -0.6987 | 4.8603 | -8.5179 |
| motor cortex _right | 932 | 9.1756 | 0.5123 | 4.9303 | -8.5179 |
| olfactory bulb_left | 109 | 7.1423 | -0.3602 | 5.084 | -4.1979 |
| olfactory bulb_right | 96 | 5.9601 | 0.0377 | 5.4079 | -4.1979 |
| olfactory tract_left | 2 | 3.9127 | -1.1034 | 6.9879 | 2.0421 |
| olfactory tract_right | 4 | 4.5938 | 0.765 | 7.0959 | 2.0421 |
| olfactory tubercle_left | 5 | 4.3119 | -2.6923 | 9.2191 | -9.4779 |
| olfactory tubercle_right | 30 | 4.6837 | 0.5014 | 7.7492 | 1.0821 |
| orbital cortex_left | 8 | 4.3708 | -1.6529 | 6.8717 | 3.0021 |
| orbital cortex_right | 30 | 4.2778 | 2.9104 | 8.2481 | -8.0379 |
| parietal association cortex_right | 11 | 4.8241 | 4.3215 | 4.0026 | -3.7179 |
| parietal cortex posterior area_left | 188 | 8.6975 | -5.4093 | 5.0781 | -5.6379 |
| parietal cortex posterior area_right | 303 | 10.2848 | 5.68 | 5.7191 | -5.6379 |
| pfi flocculonodular lobe _left | 57 | 5.7322 | -1.2769 | 6.0769 | -14.2779 |
| pfi flocculonodular lobe _right | 50 | 5.3745 | 1.2489 | 6.2229 | -14.2779 |
| pineal gland | 109 | 9.0669 | -0.4219 | 5.0356 | -8.5179 |
| piriform cortex_left | 114 | 4.9077 | -2.4419 | 8.1797 | -7.5579 |
| piriform cortex_right | 132 | 4.9804 | 2.1105 | 9.1536 | -8.9979 |
| pontine _ basilar part of pons _left | 23 | 4.4335 | -5.3895 | 5.79 | -7.0779 |
| pontine _ basilar part of pons _right | 17 | 4.8595 | 2.9434 | 8.9036 | -10.4379 |
| pontine _ tegmentum of pons_left | 806 | 5.4732 | -1.6199 | 7.2263 | 0.6021 |
| pontine _ tegmentum of pons_right | 996 | 8.717 | -0.0108 | 5.7279 | -9.4779 |
| posterior commissure_left | 12 | 4.2937 | -0.3536 | 6.7373 | -4.6779 |
| posterior commissure_right | 3 | 3.8963 | 0.4661 | 7.1278 | -5.1579 |
| prelimbic cortex_left | 43 | 7.6923 | -0.1558 | 6.1021 | -8.9979 |
| prelimbic cortex_right | 39 | 7.539 | -0.0174 | 6.1101 | -8.9979 |
| ptpretectal area _left | 22 | 7.0413 | -0.1296 | 6.4125 | -0.8379 |
| ptpretectal area _right | 18 | 4.2614 | 2.0577 | 8.0871 | -5.1579 |
| retrosplenial cortex_left | 609 | 8.6156 | -0.8371 | 4.7107 | -8.5179 |
| retrosplenial cortex_right | 517 | 7.9641 | 0.7652 | 4.4779 | -8.0379 |
| sensory cortex _left | 294 | 5.705 | -2.4132 | 3.36 | -0.8379 |
| sensory cortex _right | 347 | 6.4133 | 2.0049 | 3.6576 | -1.3179 |
| septal area _left | 537 | 6.8467 | -0.268 | 6.4045 | -0.8379 |
| septal area _right | 445 | 6.7283 | -0.0085 | 6.4195 | -0.8379 |
| striatum_left | 865 | 5.642 | -1.6199 | 7.3679 | 0.6021 |
| striatum_right | 802 | 6.3118 | 3.2293 | 6.5933 | -12.3579 |
| subthalamic nucleus_left | 1 | 3.9222 | -2.3363 | 9.4454 | -15.2379 |
| supraoptic region_left | 214 | 6.7048 | -0.1296 | 6.1293 | -0.8379 |
| supraoptic region_right | 121 | 6.3916 | 4.3347 | 7.0083 | -4.6779 |
| temporal association cortex_left | 8 | 4.8564 | -6.0642 | 6.0342 | -7.0779 |
| tenia tecta_right | 8 | 3.9483 | 1.2815 | 7.2823 | 3.4821 |
| third ventricle_left | 241 | 7.0963 | -0.2614 | 6.4471 | -1.3179 |
| third ventricle_right | 227 | 7.1909 | -0.123 | 6.4551 | -1.3179 |
| visual cortex _left | 556 | 9.3527 | -5.6795 | 4.8038 | -6.1179 |
| visual cortex _right | 451 | 10.205 | 5.6866 | 5.7617 | -6.1179 |
| prefrontal cortex _left | 57 | 7.6923 | -0.1558 | 6.1021 | -8.9979 |
| prefrontal cortex _right | 75 | 7.539 | -0.0174 | 6.1101 | -8.9979 |
| olfactory cortex_right | 27 | 5.1104 | 7.0121 | 8.7166 | -5.6379 |
| temporal association cortex_right | 11 | 4.4333 | 7.3021 | 7.6496 | -6.5979 |
| insular cortex_right | 1 | 4.1402 | 2.2025 | 4.7731 | 4.4421 |
| midbrain _ superior colliculus _right | 32 | 4.3742 | 1.6596 | 5.2836 | 4.9221 |
| orbital cortex_right | 20 | 4.4443 | 1.6596 | 5.4252 | 4.9221 |
| olfactory cortex_left | 2 | 4.0852 | -5.7544 | 5.6791 | -9.4779 |
| midbrain _ superior colliculus _left | 11 | 4.2749 | -2.8837 | 4.7623 | 4.4421 |

Notes: AD, Axial Diffusivity; SHR, model group; WKY, normal control group. *p* < 0.001, uncorrected, Cluster > 20.

**Table S2. Brain regions with increased MD values in the SHR group compared to those in the WKY group.**

| Brain regions | Ke | *t*-value | Peak MNI coordinate (mm) | | |
| --- | --- | --- | --- | --- | --- |
|  |  |  | X | Y | Z |
| Thalamus_lateral nucleus group_left | 481 | 6.9601 | -4.3433 | 7.4339 | -5.1579 |
| Thalamus_lateral nucleus group_right | 523 | 8.314 | 4.2029 | 7.3438 | -5.1579 |
| Thalamus_medial nucleus group_left | 385 | 9.3462 | -0.0042 | 5.7705 | -9.9579 |
| Thalamus_medial nucleus group_right | 139 | 7.2462 | -0.0108 | 6.312 | -9.4779 |
| Thalamus_midline nucleus group_left | 268 | 6.7756 | -0.2482 | 6.5323 | -2.2779 |
| Thalamus_midline nucleus group_right | 162 | 7.0669 | 0.0486 | 6.1113 | -13.7979 |
| accumbens nucleus_left | 193 | 5.8343 | -2.5473 | 8.9688 | -9.9579 |
| accumbens nucleus_right | 266 | 6.1582 | 0.7782 | 7.4643 | 1.0821 |
| amygdaloid body_left | 52 | 7.8457 | -0.0108 | 5.1438 | -9.4779 |
| amygdaloid body_right | 36 | 9.1143 | -0.0108 | 6.0111 | -9.4779 |
| anterior commissure_left | 53 | 5.4177 | -1.4749 | 7.7194 | 0.1221 |
| anterior commissure_right | 59 | 5.6534 | -0.0108 | 4.5597 | -9.4779 |
| anterior nucleus group _left | 50 | 6.8683 | -0.2548 | 6.2065 | -1.7979 |
| anterior nucleus group _right | 16 | 6.1079 | 0.0047 | 7.3897 | -1.7979 |
| auditory cortex_left | 71 | 8.1028 | -5.794 | 5.7244 | -6.5979 |
| auditory cortex_right | 97 | 7.9962 | 5.5548 | 6.3804 | -6.5979 |
| bed nucleus of stria terminalis _left | 94 | 6.3348 | -3.5671 | 6.9112 | -13.7979 |
| bed nucleus of stria terminalis _right | 77 | 5.7963 | 0.5212 | 8.1602 | -0.3579 |
| blood vessel _right | 2 | 3.8603 | -0.1692 | 5.8737 | 2.0421 |
| capsule_left | 128 | 6.1502 | -3.0374 | 8.3687 | -13.3179 |
| capsule_right | 124 | 5.3786 | 3.3809 | 7.5715 | -13.3179 |
| cerebellum _anterior lobe of cerebellum _left | 1633 | 9.5018 | -0.1492 | 5.7199 | -9.4779 |
| cerebellum _anterior lobe of cerebellum _right | 1716 | 9.5004 | 0.5189 | 5.1322 | -8.9979 |
| cerebellum _cerebellar nucleus_left | 329 | 8.056 | -1.4219 | 5.7254 | -13.7979 |
| cerebellum _cerebellar nucleus_right | 287 | 7.5059 | 1.5191 | 5.8954 | -13.7979 |
| cerebellum _posterior lobe of cerebellum_left | 2519 | 8.2869 | -1.1558 | 5.783 | -14.2779 |
| cerebellum _posterior lobe of cerebellum_right | 2013 | 8.267 | 0.9894 | 5.907 | -14.2779 |
| cerebral peduncle _left | 6 | 4.9935 | -2.334 | 9.1281 | -6.5979 |
| cerebral peduncle _right | 13 | 5.7182 | 3.7984 | 8.1455 | -4.6779 |
| cingulate gyrus _left | 877 | 9.5885 | -0.1492 | 5.8615 | -9.4779 |
| cingulate gyrus _right | 1107 | 9.6254 | 0.3739 | 4.9223 | -8.5179 |
| claustral layer _left | 24 | 5.1588 | -1.9297 | 6.8557 | 3.0021 |
| claustral layer _right | 5 | 4.0288 | 3.726 | 8.121 | -9.4779 |
| corpus callosum _left | 801 | 9.1848 | -5.1498 | 5.0931 | -5.6379 |
| corpus callosum _right | 758 | 10.901 | 5.5416 | 5.9943 | -5.6379 |
| dentate gyrus_left | 476 | 8.9823 | -5.4093 | 7.1136 | -5.6379 |
| dentate gyrus_right | 440 | 10.7818 | 5.2821 | 7.4484 | -5.6379 |
| dorsal peduncular cortex_right | 2 | 4.1196 | 0.62 | 7.0453 | 2.5221 |
| fourth ventricle _left | 74 | 5.7294 | -3.7385 | 8.0177 | -11.3979 |
| fourth ventricle _right | 71 | 8.6431 | 3.8974 | 9.0854 | -11.8779 |
| hippocampus_left | 1378 | 9.5289 | -5.6795 | 6.2729 | -6.1179 |
| hippocampus_right | 1276 | 11.7593 | 5.4032 | 7.1545 | -5.6379 |
| hypothalamus_preoptic region_left | 235 | 6.1394 | -0.0966 | 8.0946 | -3.2379 |
| hypothalamus_preoptic region_right | 220 | 6.1524 | 0.4001 | 8.4541 | -0.3579 |
| hypothalamus_tuberal region _left | 500 | 6.6845 | -1.0174 | 9.7381 | -14.2779 |
| hypothalamus_tuberal region _right | 371 | 5.8047 | 0.4133 | 8.8225 | -1.3179 |
| infralimbic cortex_left | 7 | 4.0827 | -0.8505 | 6.3762 | 2.5221 |
| infralimbic cortex_right | 6 | 4.1427 | -0.1758 | 6.4152 | 2.5221 |
| insular cortex_left | 39 | 5.7089 | -4.1628 | 7.665 | -9.4779 |
| insular cortex_right | 11 | 4.3488 | 3.1897 | 8.6741 | -9.4779 |
| interstitial nucleus _left | 7 | 4.06 | -2.268 | 9.1116 | -11.3979 |
| interstitial nucleus _right | 11 | 5.1368 | 2.1435 | 9.6675 | -11.3979 |
| mammillary region_left | 62 | 6.2324 | -0.09 | 8.1372 | -3.7179 |
| mammillary region_right | 47 | 5.9951 | 0.0311 | 8.1442 | -3.7179 |
| medulla oblongata_left | 950 | 6.7061 | -0.879 | 9.6045 | -14.2779 |
| medulla oblongata_right | 721 | 6.8668 | 3.0711 | 8.9532 | -10.9179 |
| midbrain _ inferior colliculus _left | 175 | 9.158 | -0.2942 | 5.6693 | -8.9979 |
| midbrain _ inferior colliculus _right | 159 | 9.0189 | 0.5189 | 5.4154 | -8.9979 |
| midbrain _ periaqueductal gray matter_left | 27 | 7.4365 | -0.1624 | 5.9179 | -8.5179 |
| midbrain _ periaqueductal gray matter_right | 13 | 7.179 | 0.0971 | 5.9329 | -8.5179 |
| midbrain _ substantia nigra_left | 9 | 4.8861 | -2.4551 | 8.8379 | -6.5979 |
| midbrain _ substantia nigra_right | 2 | 4.3287 | 1.2817 | 9.3371 | -6.5979 |
| midbrain_tegmentum of midbrain_left | 475 | 7.1522 | -2.5803 | 8.0301 | -7.5579 |
| midbrain_tegmentum of midbrain_right | 501 | 6.9504 | -0.0108 | 4.8429 | -9.4779 |
| motor cortex _left | 1221 | 9.0968 | -0.6987 | 4.8603 | -8.5179 |
| motor cortex _right | 1105 | 9.7162 | 0.5123 | 4.9303 | -8.5179 |
| olfactory bulb_left | 213 | 7.1041 | -2.5869 | 8.1291 | -7.0779 |
| olfactory bulb_right | 183 | 6.4487 | -0.0834 | 5.6841 | -4.1979 |
| olfactory tract_left | 7 | 4.4018 | -0.8767 | 7.234 | -5.6379 |
| olfactory tract_right | 7 | 4.8281 | 0.765 | 7.0959 | 2.0421 |
| olfactory tubercle_left | 30 | 5.8359 | -2.1428 | 10.2026 | -10.4379 |
| olfactory tubercle_right | 47 | 4.8387 | 2.3873 | 9.4705 | -8.9979 |
| orbital cortex_left | 134 | 5.8706 | -2.9782 | 7.5646 | -7.5579 |
| orbital cortex_right | 57 | 5.4041 | 2.9104 | 7.9472 | -8.0379 |
| parietal association cortex_left | 5 | 4.2083 | -4.5015 | 3.4926 | -3.7179 |
| parietal association cortex_right | 28 | 5.7594 | 4.3215 | 4.0026 | -3.7179 |
| parietal cortex posterior area_left | 235 | 8.6559 | -5.4093 | 5.0781 | -5.6379 |
| parietal cortex posterior area_right | 318 | 10.9586 | 5.68 | 5.7191 | -5.6379 |
| pfi flocculonodular lobe _left | 77 | 6.5769 | -3.853 | 7.7524 | -11.8779 |
| pfi flocculonodular lobe _right | 64 | 6.5583 | 1.2489 | 6.2229 | -14.2779 |
| pineal gland | 111 | 9.5119 | 0.5123 | 5.0896 | -8.5179 |
| piriform cortex_left | 268 | 7.1011 | -2.5803 | 8.1717 | -7.5579 |
| piriform cortex_right | 208 | 7.2546 | 2.2291 | 8.7506 | -7.5579 |
| pontine _ basilar part of pons _left | 68 | 5.697 | -4.1628 | 7.8243 | -9.4779 |
| pontine _ basilar part of pons _right | 38 | 6.6366 | 3.0645 | 8.9106 | -10.4379 |
| pontine _ tegmentum of pons_left | 1494 | 6.5103 | -1.1558 | 9.5885 | -14.2779 |
| pontine _ tegmentum of pons_right | 1394 | 9.4833 | -0.0108 | 5.7279 | -9.4779 |
| posterior commissure_left | 17 | 4.8968 | -0.7449 | 7.0578 | -5.1579 |
| posterior commissure_right | 8 | 4.4683 | 0.4661 | 7.1278 | -5.1579 |
| prelimbic cortex_left | 65 | 8.1474 | -0.1558 | 6.1021 | -8.9979 |
| prelimbic cortex_right | 55 | 8.1218 | -0.0174 | 6.1101 | -8.9979 |
| ptpretectal area _left | 23 | 7.5239 | -0.1296 | 6.4125 | -0.8379 |
| ptpretectal area _right | 48 | 4.7514 | 2.0577 | 8.0871 | -5.1579 |
| retrosplenial cortex_left | 662 | 8.7265 | -0.8371 | 4.7107 | -8.5179 |
| retrosplenial cortex_right | 585 | 8.6985 | 0.7652 | 4.4779 | -8.0379 |
| sensory cortex _left | 439 | 6.5414 | -2.1032 | 5.5022 | -13.3179 |
| sensory cortex _right | 533 | 6.8074 | 2.0049 | 3.6576 | -1.3179 |
| septal area _left | 640 | 7.4575 | -0.2216 | 6.2795 | -14.2779 |
| septal area _right | 494 | 7.2938 | -0.123 | 6.1719 | -1.3179 |
| striatum_left | 1182 | 6.6607 | -3.7385 | 7.5752 | -11.3979 |
| striatum_right | 1060 | 8.4514 | 4.1635 | 7.5323 | -12.3579 |
| subthalamic nucleus_left | 8 | 5.5097 | -1.9584 | 8.8965 | -3.7179 |
| subthalamic nucleus_right | 1 | 4.1821 | 1.64 | 9.1045 | -3.7179 |
| supraoptic region_left | 316 | 7.2167 | -0.1296 | 6.1293 | -0.8379 |
| supraoptic region_right | 244 | 6.7947 | 4.3347 | 7.0083 | -4.6779 |
| tenia tecta_left | 5 | 4.6823 | -1.7979 | 7.1043 | 3.4821 |
| tenia tecta_right | 13 | 4.4891 | 1.0354 | 8.9648 | -7.5579 |
| third ventricle_left | 278 | 7.6534 | -0.2614 | 6.4471 | -1.3179 |
| third ventricle_right | 270 | 7.7973 | -0.123 | 6.4551 | -1.3179 |
| visual cortex _left | 573 | 9.5056 | -5.8006 | 4.7968 | -6.1179 |
| visual cortex _right | 465 | 10.532 | 5.6866 | 5.7617 | -6.1179 |
| prefrontal cortex _left | 206 | 8.1474 | -0.1558 | 6.1021 | -8.9979 |
| prefrontal cortex _right | 120 | 8.1218 | -0.0174 | 6.1101 | -8.9979 |
| olfactory cortex_right | 110 | 7.7002 | 6.4758 | 9.5529 | -5.6379 |
| temporal association cortex_right | 51 | 6.482 | 7.3087 | 6.8072 | -7.0779 |
| olfactory cortex_left | 22 | 5.4047 | -7.4161 | 7.5817 | -5.6379 |
| temporal association cortex_left | 30 | 5.9478 | -7.4161 | 7.2985 | -5.6379 |
| frontal cortex area 3_left | 2 | 3.9347 | -3.6463 | 6.4 | -8.0379 |
| midbrain _ superior colliculus _right | 15 | 4.2665 | 1.8046 | 5.6174 | 4.4421 |
| midbrain _ superior colliculus _left | 38 | 4.8251 | -2.354 | 5.0516 | 4.9221 |

Notes: MD, Mean Diffusivity; SHR, model group; WKY, normal control group. *p* < 0.001, uncorrected, Cluster > 20.

**Table S3. Brain regions with increased RD values in the SHR group compared to those in the WKY group.**

| Brain regions | Ke | *t*-value | Peak MNI coordinate (mm) | | |
| --- | --- | --- | --- | --- | --- |
|  |  |  | X | Y | Z |
| Thalamus_lateral nucleus group_left | 539 | 7.7104 | -4.3433 | 7.4339 | -5.1579 |
| Thalamus_lateral nucleus group_right | 651 | 8.7479 | 4.0645 | 8.2031 | -5.1579 |
| Thalamus_medial nucleus group_left | 406 | 9.9806 | -0.0042 | 5.7705 | -9.9579 |
| Thalamus_medial group_right | 161 | 7.5173 | -0.0108 | 6.312 | -9.4779 |
| Thalamus_midline nucleus group_left | 278 | 7.306 | -0.2482 | 6.5323 | -2.2779 |
| Thalamus_midline nucleus group_right | 187 | 7.508 | 0.0486 | 6.1113 | -13.7979 |
| accumbens nucleus_left | 206 | 6.2695 | -2.6857 | 8.9608 | -9.9579 |
| accumbens nucleus_right | 260 | 7.0685 | 0.7782 | 7.4643 | 1.0821 |
| amygdaloid body_left | 53 | 8.2175 | -0.0108 | 5.1438 | -9.4779 |
| amygdaloid body_right | 39 | 9.4108 | -0.0108 | 6.0111 | -9.4779 |
| anterior commissure_left | 62 | 6.5282 | -2.301 | 8.6154 | -8.9979 |
| anterior commissure_right | 55 | 6.3848 | -0.0108 | 4.5597 | -9.4779 |
| anterior nucleus group _left | 50 | 7.4307 | -0.2548 | 6.2065 | -1.7979 |
| anterior nucleus group _right | 35 | 6.5834 | 0.0047 | 7.3897 | -1.7979 |
| auditory cortex_left | 72 | 8.7328 | -5.9324 | 5.9996 | -6.5979 |
| auditory cortex_right | 88 | 8.0592 | 5.5548 | 6.3804 | -6.5979 |
| bed nucleus of stria terminalis _left | 105 | 7.1089 | -3.5671 | 6.9112 | -13.7979 |
| bed nucleus of stria terminalis _right | 64 | 6.0355 | 0.5344 | 8.5463 | -1.3179 |
| blood vessel _right | 3 | 4.2211 | -0.1692 | 5.8737 | 2.0421 |
| capsule_left | 137 | 7.3451 | -3.7121 | 6.8606 | -13.3179 |
| capsule_right | 139 | 5.8793 | 2.9764 | 7.205 | -12.8379 |
| cerebellum _anterior lobe of cerebellum _left | 1915 | 10.0029 | -0.1492 | 5.7199 | -9.4779 |
| cerebellum _anterior lobe of cerebellum _right | 2048 | 9.9543 | 0.1103 | 5.7349 | -9.4779 |
| cerebellum _cerebellar nucleus_left | 347 | 9.0952 | -1.8264 | 5.6598 | -13.3179 |
| cerebellum _cerebellar nucleus_right | 356 | 8.6606 | 1.5191 | 5.8954 | -13.7979 |
| cerebellum _posterior lobe of cerebellum _left | 2836 | 9.2694 | -1.0174 | 5.791 | -14.2779 |
| cerebellum _posterior lobe of cerebellum _right | 2395 | 9.6373 | 1.1278 | 5.915 | -14.2779 |
| cerebral peduncle _left | 2 | 4.8629 | -3.952 | 7.6975 | -4.6779 |
| cerebral peduncle _right | 17 | 6.0447 | 3.7984 | 8.1455 | -4.6779 |
| cingulate gyrus _left | 1032 | 9.8926 | -0.1492 | 5.8615 | -9.4779 |
| cingulate gyrus _right | 1261 | 9.98 | -0.0108 | 5.8695 | -9.4779 |
| claustral layer _left | 22 | 5.7667 | -4.1496 | 7.0245 | -10.4379 |
| claustral layer _right | 10 | 4.6812 | 3.4665 | 8.106 | -9.4779 |
| corpus callosum _left | 907 | 9.4841 | -5.8006 | 5.6818 | -6.1179 |
| corpus callosum _right | 901 | 11.3535 | 5.5416 | 5.9943 | -5.6379 |
| dentate gyrus_left | 483 | 9.7313 | -5.4093 | 7.1136 | -5.6379 |
| dentate gyrus_right | 484 | 11.4436 | 5.2821 | 7.4484 | -5.6379 |
| dorsal peduncular cortex_right | 1 | 3.8246 | 0.62 | 7.0453 | 2.5221 |
| fourth ventricle _left | 88 | 6.5149 | -0.024 | 6.2268 | -8.5179 |
| fourth ventricle _right | 76 | 8.9303 | 3.8974 | 9.0854 | -11.8779 |
| frontal cortex area 3_left | 3 | 5.1359 | -3.6463 | 6.4 | -8.0379 |
| hippocampus_left | 1451 | 10.1434 | -5.6795 | 6.2729 | -6.1179 |
| hippocampus_right | 1350 | 12.2702 | 5.4032 | 7.1545 | -5.6379 |
| hypothalamus_preoptic region_left | 260 | 6.5804 | -0.0966 | 8.0946 | -3.2379 |
| hypothalamus_preoptic region_right | 239 | 6.7847 | 0.4067 | 8.7799 | -0.8379 |
| hypothalamus_tuberal region _left | 535 | 7.7215 | -1.0174 | 9.7381 | -14.2779 |
| hypothalamus_tuberal region _right | 407 | 6.7077 | 0.851 | 9.8461 | -14.2779 |
| infralimbic cortex_left | 7 | 4.2744 | -0.9889 | 6.3682 | 2.5221 |
| infralimbic cortex_right | 8 | 4.4313 | -0.1758 | 6.4152 | 2.5221 |
| insular cortex_left | 46 | 5.6365 | -3.4881 | 8.2881 | -9.4779 |
| insular cortex_right | 45 | 4.7968 | 3.1897 | 8.6741 | -9.4779 |
| interstitial nucleus _left | 11 | 4.1504 | -2.3891 | 9.4055 | -11.3979 |
| interstitial nucleus _right | 11 | 5.637 | 2.1435 | 9.6675 | -11.3979 |
| mammillary region_left | 60 | 7.0946 | -0.6263 | 8.1062 | -3.7179 |
| mammillary region_right | 47 | 6.39 | 0.0311 | 8.1442 | -3.7179 |
| medulla oblongata_left | 1016 | 7.7577 | -0.879 | 9.6045 | -14.2779 |
| medulla oblongata_right | 789 | 7.9061 | 2.95 | 8.9462 | -10.9179 |
| midbrain _ inferior colliculus _left | 219 | 9.4459 | -0.1558 | 5.6773 | -8.9979 |
| midbrain _ inferior colliculus _right | 182 | 9.3727 | -0.0174 | 5.6853 | -8.9979 |
| midbrain _ periaqueductal gray matter_left | 40 | 7.8041 | -0.024 | 5.9259 | -8.5179 |
| midbrain _ periaqueductal gray matter_right | 21 | 7.6885 | 0.0971 | 5.9329 | -8.5179 |
| midbrain _ substantia nigra_left | 1 | 3.8577 | -2.5935 | 8.529 | -6.5979 |
| midbrain _ substantia nigra_right | 3 | 4.5157 | 1.818 | 9.0849 | -6.5979 |
| midbrain _ superior colliculus _left | 84 | 5.4736 | -0.1756 | 5.5495 | -7.5579 |
| midbrain _ superior colliculus _right | 31 | 5.0797 | 0.0839 | 5.5645 | -7.5579 |
| midbrain_tegmentum of midbrain_left | 567 | 7.526 | 1.6336 | 5.5766 | -13.3179 |
| midbrain_tegmentum of midbrain_right | 518 | 7.5218 | -0.0108 | 4.8429 | -9.4779 |
| motor cortex _left | 1441 | 9.0739 | -0.6987 | 4.8603 | -8.5179 |
| motor cortex _right | 1332 | 10.0808 | 0.5123 | 4.9303 | -8.5179 |
| olfactory bulb_left | 323 | 7.2609 | -0.2218 | 5.3929 | -4.1979 |
| olfactory bulb_right | 283 | 6.9347 | -0.0834 | 5.6841 | -4.1979 |
| olfactory tract_left | 10 | 4.8786 | -0.8767 | 7.234 | -5.6379 |
| olfactory tract_right | 10 | 5.0152 | 0.765 | 7.0959 | 2.0421 |
| olfactory tubercle_left | 37 | 5.481 | -2.0044 | 10.2106 | -10.4379 |
| olfactory tubercle_right | 79 | 5.352 | 2.3873 | 9.4705 | -8.9979 |
| orbital cortex_left | 204 | 6.8574 | -2.9716 | 7.6072 | -8.0379 |
| orbital cortex_right | 59 | 6.6131 | 3.0315 | 7.9542 | -8.0379 |
| parietal association cortex_left | 62 | 5.3246 | -1.4021 | 4.8266 | -15.2379 |
| parietal association cortex_right | 88 | 6.3322 | 4.3215 | 4.0026 | -3.7179 |
| parietal cortex posterior area_left | 286 | 9.2926 | -5.939 | 5.6738 | -6.1179 |
| parietal cortex posterior area_right | 334 | 11.0743 | 5.5416 | 5.7111 | -5.6379 |
| pfi flocculonodular lobe _left | 93 | 7.0474 | -3.853 | 7.7524 | -11.8779 |
| pfi flocculonodular lobe _right | 103 | 7.5157 | 1.2489 | 6.2229 | -14.2779 |
| pineal gland | 112 | 9.8599 | 0.5123 | 5.0896 | -8.5179 |
| piriform cortex_left | 327 | 7.1246 | -2.4353 | 8.2223 | -8.0379 |
| piriform cortex_right | 212 | 7.0817 | 2.1105 | 9.1536 | -8.9979 |
| pontine _ basilar part of pons _left | 82 | 5.8568 | -2.82 | 9.0279 | -8.9979 |
| pontine _ basilar part of pons _right | 55 | 7.6474 | 2.9434 | 8.9036 | -10.4379 |
| pontine _ tegmentum of pons_left | 1677 | 7.4024 | -1.1624 | 9.8291 | -13.7979 |
| pontine _ tegmentum of pons_right | 1507 | 10.0319 | -0.0108 | 5.7279 | -9.4779 |
| posterior commissure_left | 21 | 5.5143 | -0.7449 | 7.0578 | -5.1579 |
| posterior commissure_right | 14 | 5.033 | 0.3277 | 7.1198 | -5.1579 |
| prelimbic cortex_left | 92 | 8.3967 | -0.1558 | 6.1021 | -8.9979 |
| prelimbic cortex_right | 73 | 8.4705 | -0.0174 | 6.1101 | -8.9979 |
| ptpretectal area _left | 28 | 7.8059 | -0.1296 | 6.4125 | -0.8379 |
| ptpretectal area _right | 59 | 5.372 | 2.1961 | 7.511 | -5.1579 |
| retrosplenial cortex_left | 715 | 8.9631 | -0.4351 | 4.3663 | -7.5579 |
| retrosplenial cortex_right | 619 | 9.2581 | 0.7652 | 4.4779 | -8.0379 |
| sensory cortex _left | 664 | 7.3219 | -2.1032 | 5.5022 | -13.3179 |
| sensory cortex _right | 900 | 7.3073 | 2.2885 | 5.6294 | -11.8779 |
| septal area _left | 672 | 8.2599 | -0.2216 | 6.2795 | -14.2779 |
| septal area _right | 552 | 7.9189 | 0.0552 | 6.2955 | -14.2779 |
| striatum_left | 1307 | 7.7045 | -3.3233 | 6.7142 | -11.3979 |
| striatum_right | 1237 | 8.7254 | 3.8974 | 8.9261 | -11.8779 |
| subthalamic nucleus_left | 8 | 5.2131 | -1.9584 | 8.8965 | -3.7179 |
| supraoptic region_left | 337 | 7.5533 | -0.1296 | 6.1293 | -0.8379 |
| supraoptic region_right | 258 | 7.057 | 4.3347 | 7.0083 | -4.6779 |
| tenia tecta_left | 4 | 4.4822 | -2.0374 | 7.9621 | -8.0379 |
| tenia tecta_right | 30 | 5.7157 | 1.0354 | 8.9648 | -7.5579 |
| third ventricle_left | 308 | 8.078 | -0.2614 | 6.4471 | -1.3179 |
| third ventricle_right | 298 | 8.2125 | -0.123 | 6.4551 | -1.3179 |
| visual cortex _left | 587 | 9.7646 | -5.5411 | 4.5286 | -6.1179 |
| visual cortex _right | 478 | 10.6998 | 5.5482 | 6.0369 | -6.1179 |
| prefrontal cortex _left | 303 | 8.3967 | -0.1558 | 6.1021 | -8.9979 |
| prefrontal cortex _right | 141 | 8.4705 | -0.0174 | 6.1101 | -8.9979 |
| olfactory cortex_left | 20 | 4.9191 | -7.4095 | 7.6243 | -6.1179 |
| temporal association cortex_left | 26 | 4.9606 | -7.4095 | 7.0402 | -6.1179 |
| olfactory cortex_right | 86 | 6.683 | 7.0187 | 8.7592 | -6.1179 |
| temporal association cortex_right | 41 | 6.3917 | 7.1769 | 6.8418 | -7.5579 |

Notes: RD, Radial Diffusivity; SHR, model group; WKY, normal control group. *p* < 0.001, uncorrected, Cluster > 20.

**Table S4. Brain regions with decreased AD values in the EA group compared to those in the SHR group.**

| Brain regions | Voxel size | *t*-value | Peak MNI coordinate (mm) | | |
| --- | --- | --- | --- | --- | --- |
|  |  |  | X | Y | Z |
| amygdaloid body_right | 1 | 3.8362 | 6.3547 | 9.8468 | -5.6379 |
| olfactory cortex_right | 18 | 4.1674 | 6.6142 | 9.2777 | -5.6379 |
| accumbens nucleus_right | 4 | 4.1552 | 3.1829 | 8.7715 | 1.0821 |
| frontal association cortex_right | 2 | 4.0665 | 3.0379 | 8.4377 | 1.5621 |
| insular cortex_right | 3 | 3.8762 | 3.0445 | 8.4803 | 1.0821 |
| olfactory tubercle_right | 1 | 3.7898 | 3.3213 | 9.0804 | 1.0821 |
| piriform cortex_right | 18 | 4.1938 | 3.1763 | 8.4457 | 1.5621 |
| pontine _ tegmentum of pons_right | 45 | 4.2567 | 3.3213 | 8.6379 | 1.0821 |
| striatum_right | 12 | 4.205 | 3.3213 | 8.4963 | 1.0821 |
| tenia tecta_right | 4 | 3.8891 | 1.1631 | 9.3153 | -8.0379 |
| Thalamus_lateral nucleus group_left | 4 | 3.877 | -3.1626 | 8.4459 | -14.2779 |
| capsule_left | 9 | 3.9215 | -3.4287 | 8.6715 | -13.7979 |
| medulla oblongata_left | 6 | 4.1015 | -3.4419 | 9.0288 | -12.8379 |
| pontine _ tegmentum of pons_left | 13 | 3.9434 | -3.5737 | 8.7802 | -13.3179 |
| striatum_left | 12 | 4.1301 | -3.5803 | 8.8792 | -12.8379 |
| fourth ventricle _left | 1 | 3.8131 | -4.1562 | 8.1501 | -9.9579 |
| insular cortex_left | 12 | 4.2402 | -4.1628 | 8.2491 | -9.4779 |
| pontine _ basilar part of pons _left | 9 | 4.2597 | -4.1628 | 8.1075 | -9.4779 |
| midbrain_tegmentum of midbrain_left | 1 | 3.938 | 3.0249 | 7.77 | -7.5579 |
| midbrain_tegmentum of midbrain_right | 9 | 4.2874 | 3.1699 | 8.1215 | -8.0379 |
| orbital cortex_right | 25 | 4.3872 | 3.1699 | 7.9622 | -8.0379 |
| prefrontal cortex _right | 25 | 4.3872 | 3.1699 | 7.9622 | -8.0379 |
| sensory cortex _left | 16 | 4.0487 | -6.5345 | 6.2876 | -11.8779 |
| cerebellum _anterior lobe of cerebellum _left | 4 | 3.9557 | -4.1694 | 6.6135 | -8.9979 |
| cerebellum _posterior lobe of cerebellum_left | 5 | 3.855 | -4.5541 | 6.6757 | -9.9579 |
| olfactory cortex_left | 4 | 3.991 | -3.8992 | 6.5869 | -8.5179 |
| hippocampus_right | 1 | 3.8283 | -0.5186 | 4.6457 | 7.3221 |
| midbrain _ superior colliculus _left | 3 | 3.9434 | -0.7649 | 5.0168 | 6.3621 |
| olfactory bulb_left | 25 | 4.034 | -0.6397 | 5.0812 | 7.3221 |
| retrosplenial cortex_left | 3 | 3.8276 | -0.6265 | 4.7239 | 6.3621 |
| third ventricle_left | 6 | 4.0808 | -0.5186 | 4.9466 | 7.3221 |
| third ventricle_right | 1 | 3.9113 | -0.3802 | 4.9546 | 7.3221 |
| hippocampus_left | 11 | 3.98 | -0.3295 | 6.6409 | -15.2379 |

Notes: AD, Axial Diffusivity; EA, electroacupuncture group; SHR, model group. *p* < 0.001, uncorrected, Cluster > 20.

**Table S5. Brain regions with decreased MD values in the EA group compared to those in the SHR group.**

| Brain regions | Ke | *t*-value | Peak MNI coordinate (mm) | | |
| --- | --- | --- | --- | --- | --- |
|  |  |  | X | Y | Z |
| accumbens nucleus_left | 2 | 3.8851 | -3.3738 | 8.3925 | 1.0821 |
| amygdaloid body_left | 15 | 4.1761 | -4.1432 | 9.101 | -0.8379 |
| olfactory tubercle_left | 4 | 4.2501 | -3.9101 | 8.6624 | 1.0821 |
| piriform cortex_left | 50 | 4.4373 | -4.0246 | 8.698 | 0.6021 |
| hypothalamus_preoptic region_left | 9 | 4.0269 | -0.7909 | 10.4074 | -11.8779 |
| pontine _ tegmentum of pons_left | 30 | 4.4958 | -0.8922 | 10.3866 | -13.3179 |
| supraoptic region_left | 15 | 4.3905 | -0.8922 | 10.245 | -13.3179 |
| midbrain_tegmentum of midbrain_right | 11 | 4.1659 | 0.4991 | 9.3763 | -7.5579 |
| olfactory tubercle_right | 8 | 3.9465 | 1.0486 | 9.6341 | -8.5179 |
| pontine _ tegmentum of pons_right | 58 | 4.3022 | 0.5057 | 9.7198 | -8.0379 |
| tenia tecta_right | 10 | 3.9538 | 0.7652 | 9.2923 | -8.0379 |
| amygdaloid body_right | 2 | 5.5101 | 6.3547 | 9.8468 | -5.6379 |
| olfactory cortex_right | 39 | 6.1041 | 6.3481 | 9.8042 | -5.1579 |
| piriform cortex_right | 11 | 4.5826 | 3.5808 | 8.5113 | 1.0821 |
| striatum_right | 13 | 4.4343 | 3.4424 | 8.5033 | 1.0821 |
| medulla oblongata_left | 11 | 3.9765 | -1.1758 | 8.7157 | -2.7579 |
| Thalamus_lateral nucleus group_right | 1 | 3.8087 | 3.1631 | 7.6171 | 2.5221 |
| pontine _ basilar part of pons _right | 2 | 3.8478 | 3.4226 | 7.3489 | 2.5221 |
| claustral layer _right | 1 | 3.8856 | 3.3281 | 8.3989 | -9.4779 |
| insular cortex_right | 16 | 4.2027 | 3.1699 | 7.679 | -8.0379 |
| orbital cortex_right | 26 | 4.5722 | 3.1699 | 7.9622 | -8.0379 |
| prefrontal cortex _right | 26 | 4.5722 | 3.1699 | 7.9622 | -8.0379 |
| Thalamus_lateral nucleus group_left | 52 | 4.722 | -3.4221 | 7.8468 | -14.2779 |
| capsule_left | 10 | 4.5765 | -3.5671 | 8.0794 | -13.7979 |
| cerebellum _cerebellar nucleus_left | 18 | 4.7097 | -3.5671 | 7.6546 | -13.7979 |
| claustral layer _left | 3 | 3.9688 | -4.0178 | 8.2997 | -9.9579 |
| fourth ventricle _left | 2 | 4.2827 | -4.1562 | 8.1501 | -9.9579 |
| pontine _ basilar part of pons _left | 14 | 5.2341 | -4.1628 | 8.1075 | -9.4779 |
| pfi flocculonodular lobe _right | 8 | 4.7098 | 4.97 | 8.5633 | -11.8779 |
| olfactory cortex_left | 8 | 4.0389 | -6.2026 | 7.7785 | -7.0779 |
| pfi flocculonodular lobe _left | 11 | 4.5275 | -5.9982 | 7.9293 | -11.8779 |
| auditory cortex_right | 11 | 4.5507 | 7.0055 | 7.5058 | -5.1579 |
| temporal association cortex_right | 14 | 4.7201 | 7.0055 | 7.789 | -5.1579 |
| corpus callosum _right | 3 | 4.1855 | 5.568 | 7.6338 | -7.5579 |
| Thalamus_midline nucleus group_left | 5 | 3.9694 | -1.011 | 7.0002 | -4.6779 |
| anterior commissure_left | 4 | 4.3124 | -1.1362 | 7.219 | -5.6379 |
| hypothalamus_tuberal region _left | 10 | 4.1838 | -1.0044 | 7.0428 | -5.1579 |
| midbrain _ periaqueductal gray matter_left | 11 | 4.0793 | -0.8767 | 7.3756 | -5.6379 |
| midbrain _ superior colliculus _left | 23 | 4.1956 | -1.1362 | 7.0774 | -5.6379 |
| midbrain_tegmentum of midbrain_left | 28 | 4.2028 | -1.1362 | 7.3606 | -5.6379 |
| olfactory bulb_left | 71 | 4.3012 | -1.1428 | 7.1764 | -5.1579 |
| olfactory tract_left | 6 | 4.1403 | -0.8767 | 7.234 | -5.6379 |
| posterior commissure_left | 2 | 3.9561 | -0.7449 | 7.0578 | -5.1579 |
| ptpretectal area _left | 6 | 4.0335 | -1.8175 | 7.279 | -5.1579 |
| frontal cortex area 3_left | 4 | 4.0107 | -3.8047 | 5.5369 | 3.4821 |
| insular cortex_left | 23 | 4.1893 | -3.6729 | 5.5023 | 3.9621 |
| midbrain _ inferior colliculus _left | 5 | 3.8945 | -3.1366 | 5.6749 | 3.9621 |
| sensory cortex _right | 10 | 4.0432 | 6.8209 | 7.4828 | -1.7979 |
| cerebellum _anterior lobe of cerebellum _left | 13 | 4.6914 | -4.5607 | 6.6331 | -9.4779 |
| cerebellum _posterior lobe of cerebellum_left | 29 | 4.6791 | -4.5541 | 6.6757 | -9.9579 |
| sensory cortex _left | 61 | 4.7507 | -4.5607 | 6.7747 | -9.4779 |
| cerebellum _anterior lobe of cerebellum _right | 8 | 3.948 | -3.2022 | 6.8805 | -11.3979 |
| striatum_left | 36 | 3.9438 | -3.2022 | 7.0221 | -11.3979 |
| frontal association cortex_left | 2 | 3.8846 | -0.7583 | 4.9178 | 5.8821 |
| orbital cortex_left | 13 | 4.1059 | -1.1496 | 5.5215 | 5.4021 |
| retrosplenial cortex_left | 7 | 4.0443 | -0.7649 | 4.7159 | 6.3621 |
| prefrontal cortex _left | 13 | 4.1059 | -1.1496 | 5.5215 | 5.4021 |
| auditory cortex_left | 27 | 4.2265 | -7.5545 | 5.8214 | -5.6379 |
| cerebellum _posterior lobe of cerebellum_right | 8 | 4.1757 | 4.4271 | 7.0206 | -11.3979 |
| dentate gyrus_left | 23 | 4.3824 | -2.0768 | 6.2567 | -15.2379 |
| hippocampus_left | 14 | 4.4281 | -2.2152 | 5.9478 | -15.2379 |
| frontal cortex area 3_right | 3 | 3.9983 | 3.9764 | 6.5152 | -7.5579 |
| hippocampus_right | 11 | 4.068 | 4.1041 | 6.4232 | -8.0379 |
| motor cortex _right | 6 | 4.0404 | 4.1041 | 6.2639 | -8.0379 |
| olfactory bulb_right | 11 | 4.241 | 1.3564 | 4.9556 | 6.8421 |
| corpus callosum _left | 4 | 3.9593 | -1.4021 | 5.4107 | -15.2379 |
| motor cortex _left | 8 | 4.5519 | -1.5471 | 4.776 | -14.7579 |
| parietal association cortex_left | 4 | 5.3376 | -1.4021 | 4.8266 | -15.2379 |

Notes: MD, Mean Diffusivity; EA, electroacupuncture group; SHR, model group. *p* < 0.001, uncorrected, Cluster > 20.

**Table S6. Brain regions with decreased RD values in the EA group compared to those in the SHR group.**

| Brain regions | Ke | *t*-value | Peak MNI coordinate (mm) | | | |
| --- | --- | --- | --- | --- | --- | --- |
|  |  |  | X | Y | Z | |
| amygdaloid body_right | 25 | 4.4047 | 5.1305 | 9.9748 | | -4.6779 |
| supraoptic region_left | 12 | 4.2255 | -0.8922 | 10.245 | | -13.3179 |
| olfactory tubercle_left | 6 | 4.2589 | -0.8107 | 9.9964 | | -10.4379 |
| hypothalamus_preoptic region_right | 2 | 3.8501 | 2.528 | 9.4444 | | -0.3579 |
| medulla oblongata_right | 5 | 4.904 | 2.6598 | 9.2682 | | 0.1221 |
| striatum_right | 20 | 5.9883 | 2.5214 | 9.4018 | | 0.1221 |
| accumbens nucleus_left | 4 | 4.4702 | -3.4949 | 8.3855 | | 1.0821 |
| piriform cortex_left | 13 | 4.542 | -3.6333 | 8.3775 | | 1.0821 |
| pontine _ tegmentum of pons_left | 41 | 5.0353 | -3.4883 | 8.5697 | | 0.6021 |
| amygdaloid body_left | 11 | 4.4333 | -4.275 | 8.8524 | | -1.3179 |
| interstitial nucleus _left | 4 | 3.9635 | -4.275 | 8.5515 | | -1.3179 |
| accumbens nucleus_right | 3 | 3.9363 | 0.7982 | 9.5053 | | -10.4379 |
| midbrain_tegmentum of midbrain_right | 15 | 4.0163 | 0.5123 | 9.4615 | | -8.5179 |
| olfactory tubercle_right | 21 | 4.2677 | 0.6532 | 9.7379 | | -9.9579 |
| pontine _ tegmentum of pons_right | 78 | 4.2444 | 0.6532 | 9.5963 | | -9.9579 |
| tenia tecta_right | 9 | 3.8684 | 0.7652 | 9.2923 | | -8.0379 |
| Thalamus_medial nucleus group_left | 1 | 3.8601 | -0.1096 | 9.3208 | | -12.3579 |
| hypothalamus_preoptic region_left | 15 | 4.2091 | -0.248 | 9.6137 | | -12.3579 |
| bed nucleus of stria terminalis _left | 10 | 4.065 | -1.2022 | 7.8196 | | -0.8379 |
| hypothalamus_tuberal region _left | 21 | 4.2012 | -1.1758 | 8.5741 | | -2.7579 |
| medulla oblongata_left | 32 | 4.1008 | -1.1758 | 8.7157 | | -2.7579 |
| Thalamus_lateral nucleus group_right | 3 | 4.3748 | 2.6268 | 7.887 | | 2.5221 |
| piriform cortex_right | 4 | 4.1821 | 2.8929 | 8.0862 | | 2.0421 |
| fourth ventricle _right | 8 | 4.03 | 3.8974 | 9.0854 | | -11.8779 |
| pfi flocculonodular lobe _right | 6 | 4.0985 | 3.7722 | 8.8617 | | -12.8379 |
| Thalamus_lateral nucleus group_right | 3 | 4.5883 | 3.3215 | 8.4979 | | -8.9979 |
| cerebellum _anterior lobe of cerebellum _right | 17 | 4.7325 | 3.1831 | 8.2067 | | -8.9979 |
| claustral layer _right | 6 | 4.3138 | 3.3281 | 8.3989 | | -9.4779 |
| insular cortex_right | 37 | 5.1703 | 3.1699 | 7.679 | | -8.0379 |
| orbital cortex_right | 24 | 5.3873 | 3.1699 | 7.9622 | | -8.0379 |
| pontine _ basilar part of pons _right | 12 | 4.7884 | 3.4599 | 8.5059 | | -8.9979 |
| prefrontal cortex _right | 24 | 5.3873 | 3.1699 | 7.9622 | | -8.0379 |
| pfi flocculonodular lobe _left | 30 | 4.7701 | -5.9982 | 7.9293 | | -11.8779 |
| capsule_left | 3 | 4.0832 | -3.5671 | 8.0794 | | -13.7979 |
| cerebellum _cerebellar nucleus_left | 13 | 4.1602 | -3.8332 | 7.597 | | -13.3179 |
| Thalamus_lateral nucleus group_left | 69 | 4.2012 | -3.0244 | 8.0098 | | -4.1979 |
| Thalamus_midline nucleus group_left | 23 | 4.403 | -1.1494 | 7.2754 | | -4.6779 |
| anterior commissure_left | 10 | 4.7697 | -0.9978 | 7.227 | | -5.6379 |
| midbrain _ superior colliculus _left | 41 | 4.5425 | -1.2746 | 7.0694 | | -5.6379 |
| midbrain_tegmentum of midbrain_left | 48 | 4.7485 | -0.9978 | 7.3686 | | -5.6379 |
| olfactory bulb_left | 179 | 4.7424 | -1.0044 | 7.1844 | | -5.1579 |
| olfactory tract_left | 7 | 4.7519 | -0.8767 | 7.234 | | -5.6379 |
| posterior commissure_left | 4 | 4.3907 | -0.7449 | 7.0578 | | -5.1579 |
| ptpretectal area _left | 9 | 4.1209 | -1.8175 | 7.279 | | -5.1579 |
| auditory cortex_right | 24 | 5.1386 | 7.0055 | 7.5058 | | -5.1579 |
| temporal association cortex_right | 11 | 4.7626 | 7.0055 | 7.789 | | -5.1579 |
| ptpretectal area _right | 16 | 4.3462 | 2.3345 | 7.519 | | -5.1579 |
| corpus callosum _right | 23 | 4.8889 | 5.0317 | 6.7178 | | -7.5579 |
| frontal cortex area 3_right | 14 | 4.8276 | 5.1767 | 7.2109 | | -8.0379 |
| motor cortex _right | 10 | 4.9367 | 5.1767 | 6.6268 | | -8.0379 |
| olfactory cortex_right | 122 | 6.4168 | 5.4362 | 7.3675 | | -8.0379 |
| cerebellum _anterior lobe of cerebellum _left | 37 | 4.0399 | -4.2839 | 6.6491 | | -9.4779 |
| cerebellum _posterior lobe of cerebellum _left | 79 | 4.1399 | -3.0704 | 6.2618 | | -10.9179 |
| claustral layer _left | 4 | 4.074 | -4.1562 | 8.0085 | | -9.9579 |
| fourth ventricle _left | 4 | 4.5142 | -4.1562 | 8.1501 | | -9.9579 |
| insular cortex_left | 43 | 5.5122 | -4.1628 | 7.9659 | | -9.4779 |
| olfactory cortex_left | 3 | 4.2793 | -3.7781 | 6.5939 | | -8.5179 |
| pontine _ basilar part of pons _left | 24 | 6.894 | -4.1628 | 8.1075 | | -9.4779 |
| striatum_left | 89 | 4.1446 | -3.2088 | 6.3954 | | -10.9179 |
| midbrain _ inferior colliculus _left | 7 | 3.9678 | -1.9429 | 5.7439 | | 3.9621 |
| orbital cortex_left | 7 | 4.0562 | -1.9429 | 5.8855 | | 3.9621 |
| prefrontal cortex _left | 7 | 4.0562 | -1.9429 | 5.8855 | | 3.9621 |
| frontal cortex area 3_left | 5 | 4.3167 | -3.6663 | 5.5449 | | 3.4821 |
| Thalamus_lateral nucleus group_right | 35 | 4.2907 | 2.561 | 6.4537 | | -2.7579 |
| cerebellum _cerebellar nucleus_right | 31 | 4.181 | 2.5544 | 6.2518 | | -2.2779 |
| septal area _right | 16 | 4.0229 | 2.4333 | 6.1032 | | -2.2779 |
| third ventricle_right | 6 | 3.9126 | 2.8246 | 5.8004 | | -1.7979 |
| dentate gyrus_left | 21 | 4.2396 | -1.9318 | 6.5905 | | -15.7179 |
| hippocampus_left | 22 | 6.176 | -1.9384 | 5.6806 | | -15.2379 |
| parietal association cortex_left | 5 | 5.0393 | -1.5405 | 5.1195 | | -15.2379 |
| septal area _left | 2 | 4.2842 | -1.6855 | 5.653 | | -14.7579 |
| corpus callosum _left | 16 | 4.2299 | -0.6331 | 4.3981 | | 6.8421 |
| frontal association cortex_left | 2 | 3.8444 | -0.8967 | 4.9098 | | 5.8821 |
| retrosplenial cortex_left | 21 | 4.203 | -0.7649 | 4.7159 | | 6.3621 |
| cerebellum _posterior lobe of cerebellum _right | 75 | 4.5887 | 4.97 | 6.5101 | | -11.8779 |
| sensory cortex _right | 87 | 4.6944 | 4.97 | 6.6517 | | -11.8779 |
| hippocampus_right | 14 | 4.4358 | 1.3498 | 4.7537 | | 7.3221 |
| midbrain _ superior colliculus _right | 4 | 4.369 | 1.2419 | 5.1328 | | 6.3621 |
| olfactory bulb_right | 17 | 4.7559 | 1.3564 | 4.9556 | | 6.8421 |
| sensory cortex _left | 68 | 4.551 | -6.2643 | 5.9601 | | -11.3979 |
| cingulate gyrus _left | 4 | 3.9984 | -1.3297 | 5.2936 | | -10.4379 |
| motor cortex _left | 14 | 4.0655 | -1.4681 | 5.2856 | | -10.4379 |

Notes: RD, Radial Diffusivity; EA, electroacupuncture group; SHR, model group. p < 0.001, uncorrected, Cluster > 20.

**Table S7. The callback brain regions of the AD value.**

| Number | Callback brain regions |
| --- | --- |
| 1 | amygdaloid body_right |
| 2 | olfactory cortex_right |
| 3 | accumbens nucleus_right |
| 4 | insular cortex_right |
| 5 | olfactory tubercle_right |
| 6 | piriform cortex_right |
| 7 | pontine _ tegmentum of pons_right |
| 8 | striatum_left |
| 9 | striatum_right |
| 10 | midbrain_tegmentum of midbrain_right |
| 11 | tenia tecta_right |
| 12 | Thalamus_lateral nucleus group_left |
| 13 | capsule_left |
| 14 | medulla oblongata_left |
| 15 | pontine _ tegmentum of pons_left |
| 16 | fourth ventricle _left |
| 17 | insular cortex_left |
| 18 | pontine _ basilar part of pons _left |
| 19 | midbrain_tegmentum of midbrain_left |
| 20 | orbital cortex_right |
| 21 | prefrontal cortex _right |
| 22 | sensory cortex _left |
| 23 | cerebellum _anterior lobe of cerebellum _left |
| 24 | cerebellum _posterior lobe of cerebellum_left |
| 25 | olfactory cortex_left |
| 26 | hippocampus_right |
| 27 | midbrain _ superior colliculus _left |
| 28 | olfactory bulb_left |
| 29 | retrosplenial cortex_left |
| 30 | third ventricle_left |
| 31 | third ventricle_right |
| 32 | hippocampus_left |

Notes: AD, Axial Diffusivity.

**Table S8. The callback brain regions of the MD value.**

| Number | Callback brain regions |
| --- | --- |
| 1 | amygdaloid body_left |
| 2 | olfactory tubercle_left |
| 3 | piriform cortex_left |
| 4 | pontine _ tegmentum of pons_left |
| 5 | striatum_left |
| 6 | Thalamus_lateral nucleus group_left |
| 7 | Thalamus_lateral nucleus group_right |
| 8 | Thalamus_midline nucleus group_left |
| 9 | accumbens nucleus_left |
| 10 | amygdaloid body_right |
| 11 | anterior commissure_left |
| 12 | auditory cortex_left |
| 13 | auditory cortex_right |
| 14 | capsule_left |
| 15 | cerebellum _anterior lobe of cerebellum _left |
| 16 | cerebellum _anterior lobe of cerebellum _right |
| 17 | cerebellum _cerebellar nucleus_left |
| 18 | cerebellum _posterior lobe of cerebellum _left |
| 19 | hypothalamus_preoptic region_left |
| 20 | hypothalamus_tuberal region _left |
| 21 | olfactory tubercle_right |
| 22 | supraoptic region_left |
| 23 | cerebellum _posterior lobe of cerebellum _right |
| 24 | claustral layer _left |
| 25 | claustral layer _right |
| 26 | corpus callosum _left |
| 27 | corpus callosum _right |
| 28 | dentate gyrus_left |
| 29 | fourth ventricle _left |
| 30 | hippocampus_left |
| 31 | hippocampus_right |
| 32 | insular cortex_left |
| 33 | insular cortex_right |
| 34 | medulla oblongata_left |
| 35 | midbrain _ inferior colliculus _left |
| 36 | midbrain _ periaqueductal gray matter_left |
| 37 | midbrain _ superior colliculus _left |
| 38 | midbrain_tegmentum of midbrain_left |
| 39 | midbrain_tegmentum of midbrain_right |
| 40 | motor cortex _left |
| 41 | motor cortex _right |
| 42 | olfactory bulb_left |
| 43 | olfactory bulb_right |
| 44 | pontine _ basilar part of pons _right |
| 45 | pontine _ tegmentum of pons_right |
| 46 | striatum_right |
| 47 | olfactory tract_left |
| 48 | pontine _ basilar part of pons _left |
| 49 | orbital cortex_left |
| 50 | orbital cortex_right |
| 51 | parietal association cortex_left |
| 52 | pfi flocculonodular lobe _left |
| 53 | pfi flocculonodular lobe _right |
| 54 | piriform cortex_right |
| 55 | posterior commissure_left |
| 56 | ptpretectal area _left |
| 57 | retrosplenial cortex_left |
| 58 | sensory cortex _left |
| 59 | sensory cortex _right |
| 60 | temporal association cortex_right |
| 61 | tenia tecta_right |
| 62 | prefrontal cortex _left |
| 63 | prefrontal cortex _right |
| 64 | olfactory cortex_left |

Notes: MD, Mean Diffusivity.

**Table S9. The callback brain regions of the RD value.**

| Number | Callback brain regions |
| --- | --- |
| 1 | Thalamus_lateral nucleus group_left |
| 2 | Thalamus_lateral nucleus group_right |
| 3 | Thalamus_medial nucleus group_left |
| 4 | Thalamus_midline nucleus group_left |
| 5 | accumbens nucleus_left |
| 6 | accumbens nucleus_right |
| 7 | amygdaloid body_left |
| 8 | amygdaloid body_right |
| 9 | anterior commissure_left |
| 10 | auditory cortex_right |
| 11 | bed nucleus of stria terminalis _left |
| 12 | capsule_left |
| 13 | cerebellum _anterior lobe of cerebellum _left |
| 14 | cerebellum _anterior lobe of cerebellum _right |
| 15 | cerebellum _cerebellar nucleus_left |
| 16 | cerebellum _cerebellar nucleus_right |
| 17 | cerebellum _posterior lobe of cerebellum _left |
| 18 | cerebellum _posterior lobe of cerebellum _right |
| 19 | cingulate gyrus _left |
| 20 | claustral layer _left |
| 21 | claustral layer _right |
| 22 | corpus callosum _left |
| 23 | corpus callosum _right |
| 24 | dentate gyrus_left |
| 25 | fourth ventricle _left |
| 26 | fourth ventricle _right |
| 27 | frontal cortex area 3_left |
| 28 | hippocampus_left |
| 29 | hippocampus_right |
| 30 | hypothalamus_preoptic region_left |
| 31 | hypothalamus_preoptic region_right |
| 32 | hypothalamus_tuberal region _left |
| 33 | insular cortex_left |
| 34 | insular cortex_right |
| 35 | interstitial nucleus _left |
| 36 | medulla oblongata_left |
| 37 | medulla oblongata_right |
| 38 | midbrain _ inferior colliculus _left |
| 39 | midbrain _ periaqueductal gray matter_left |
| 40 | midbrain _ superior colliculus _left |
| 41 | midbrain _ superior colliculus _right |
| 42 | midbrain_tegmentum of midbrain_left |
| 43 | midbrain_tegmentum of midbrain_right |
| 44 | motor cortex _left |
| 45 | motor cortex _right |
| 46 | olfactory bulb_left |
| 47 | olfactory bulb_right |
| 48 | olfactory cortex_left |
| 49 | olfactory cortex_right |
| 50 | olfactory tract_left |
| 51 | olfactory tubercle_left |
| 52 | olfactory tubercle_right |
| 53 | orbital cortex_left |
| 54 | orbital cortex_right |
| 55 | parietal association cortex_left |
| 56 | pfi flocculonodular lobe _left |
| 57 | pfi flocculonodular lobe _right |
| 58 | piriform cortex_left |
| 59 | piriform cortex_right |
| 60 | pontine _ basilar part of pons _left |
| 61 | pontine _ basilar part of pons _right |
| 62 | pontine _ tegmentum of pons_left |
| 63 | pontine _ tegmentum of pons_right |
| 64 | posterior commissure_left |
| 65 | ptpretectal area _left |
| 66 | ptpretectal area _right |
| 67 | retrosplenial cortex_left |
| 68 | sensory cortex _left |
| 69 | sensory cortex _right |
| 70 | septal area _left |
| 71 | septal area _right |
| 72 | striatum_left |
| 73 | striatum_right |
| 74 | supraoptic region_left |
| 75 | temporal association cortex_left |
| 76 | temporal association cortex_right |
| 77 | tenia tecta_right |
| 78 | third ventricle_right |
| 79 | prefrontal cortex _left |
| 80 | prefrontal cortex _right |

Notes: RD, Radial Diffusivity.

**Table S10. The callback brain regions common to AD, MD, and RD values.**

| Number | Callback brain regions |
| --- | --- |
| 1 | amygdaloid body_right |
| 2 | insular cortex_right |
| 3 | olfactory tubercle_right |
| 4 | piriform cortex_right |
| 5 | pontine _ tegmentum of pons_right |
| 6 | striatum_left |
| 7 | striatum_right |
| 8 | midbrain_tegmentum of midbrain_right |
| 9 | tenia tecta_right |
| 10 | Thalamus_lateral nucleus group_left |
| 11 | capsule_left |
| 12 | medulla oblongata_left |
| 13 | pontine _ tegmentum of pons_left |
| 14 | fourth ventricle _left |
| 15 | insular cortex_left |
| 16 | pontine _ basilar part of pons _left |
| 17 | midbrain_tegmentum of midbrain_left |
| 18 | orbital cortex_right |
| 19 | prefrontal cortex _right |
| 20 | sensory cortex _left |
| 21 | cerebellum _anterior lobe of cerebellum _left |
| 22 | cerebellum _posterior lobe of cerebellum _left |
| 23 | olfactory cortex_left |
| 24 | hippocampus_right |
| 25 | midbrain _ superior colliculus _left |
| 26 | olfactory bulb_left |
| 27 | retrosplenial cortex_left |
| 28 | hippocampus_left |

Notes: AD, Axial Diffusivity； MD, Mean Diffusivity；RD, Radial Diffusivity.
